# Observational study on fluid therapy management in surgical adult patients

**DOI:** 10.1186/s12871-021-01518-z

**Published:** 2021-12-13

**Authors:** Maria J. Colomina, Javier Ripollés-Melchor, Patricia Guilabert, José Luis Jover, Misericordia Basora, Concha Cassinello, Raquel Ferrandis, Juan V. Llau, Judith Peñafiel

**Affiliations:** 1 Department of Anesthesia, Critical care and Pain Clinic, Hospital Universitari de Bellvitge, Barcelona, Spain; 2 Barcelona University, Barcelona, Spain; 3 Bellvitge Research Institute, IDIBELL, Barcelona, Spain; 4 Department of Anaesthesia, Hospital Universitario Infanta Leonor, Madrid, Spain; 5 Department of Anaesthesia, Hospital Universitari Vall d’Hebron, Barcelona, Spain; 6 Department of Anaesthesia, Hospital Verge dels Lliris, Alcoi, Alicante, Spain; 7 Department of Anaesthesia, Hospital Clínic, Barcelona, Spain; 8 Department of Anaesthesia, Hospital Universitario Miguel Servet, Zaragoza, Spain; 9 Department of Anaesthesia, Hospital Universitari Politèncic La Fe, Valencia, Spain; 10 Department of Anaesthesia, Hospital Universitari Dr. Peset, València, Spain; 11 Biostatistics Unit, Bellvitge University Hospital, Barcelona, Spain

**Keywords:** Fluid therapy, Surgical procedures, Practice guidelines, Fluid therapy management, Balanced crystalloids, Hemodynamic monitoring

## Abstract

**Background:**

Perioperative fluid therapy management is changing due to the incorporation of different fluids, surgical techniques, and minimally invasive monitoring systems. The objective of this study was to explore fluid therapy management during the perioperative period in our country.

**Methods:**

We designed the *Fluid Day study* as a cross-sectional, multicentre, observational study. The study was performed in 131 Spanish hospitals in February 2019. We included adult patients undergoing general anaesthesia for either elective or non-elective surgery. Demographic variables were recorded, as well as the type and total volume of fluid administered during the perioperative period and the monitorization used. To perform the analysis, patients were categorized by risk group.

**Results:**

We recruited 7291 patients, 6314 of which were included in the analysis; 1541 (24.4%) patients underwent high-risk surgery, 1497 (23. 7%) were high risk patients, and 554 (8.7%) were high-risk patients and underwent high-risk surgery; 98% patients received crystalloids (80% balanced solutions); intraoperative colloids were used in 466 patients (7.51%). The hourly intraoperative volume in mL/kg/h and the median [Q1; Q3] administered volume (mL/kg) were, respectively, 6.67 [3.83; 8.17] ml/Kg/h and 13.9 [9.52;5.20] ml/Kg in low-risk patients undergoing low- or intermediate-risk surgery, 6 [4.04; 9.08] ml/Kg/h and 15.7 [10.4;24.5] ml/Kg in high- risk patients undergoing low or intermediate-risk surgery, 6.41 [4.36; 9.33] ml/Kg/h and 20.2 [13.3;32.4] ml/Kg in low-risk patients undergoing high-risk surgery, and 5.46 [3.83; 8.17] ml/Kg/h and 22.7[14.1;40.9] ml/Kg in high-risk patients undergoing high- risk surgery . We used advanced fluid monitoring strategies in 5% of patients in the intraoperative period and in 10% in the postoperative period.

**Conclusions:**

The most widely used fluid was balanced crystalloids. Colloids were used in a small number of patients. Hourly surgery volume tended to be more restrictive in high-risk patients but confirms a high degree of variation in the perioperatively administered volume. Scarce monitorization was observed in fluid therapy management.

**Trial registration:**

Clinical Trials: NCT03630744.

**Supplementary Information:**

The online version contains supplementary material available at 10.1186/s12871-021-01518-z.

## Background

The goal of perioperative fluid therapy is to maintain the body in an optimal state of tissue perfusion and hydration, ensuring adequate hydro-electrolytic homeostasis to provide a correct balance between oxygen tissue supply and demand, avoiding adverse side effects [1–3].

Although the use of intravenous fluids is one of the most frequent interventions in the perioperative period of any surgical scenario, the choice between the different fluids, their dosage, management, and monitoring, remains controversial [4–6]. Fluids should be administered according to therapeutic targets, and they should be given at the right time and dosage, respecting contraindications, considering the clinical status of the patient and the type of surgery performed [2, 3, 6–8]. Incorrect management of perioperative fluid therapy has been shown to have important repercussions in the immediate postoperative period, especially in highly complex patients and surgeries [9].

Several studies have investigated fluid therapy management in critically ill patients and perioperative fluid management; however, very few studies have examined whether published recommendations are followed in clinical practice [8].

Perioperative fluid therapy management varies greatly, and this has a significant factor on the postoperative evolution of patients, particularly highly complex patients, and high-complexity surgeries [9]. In a study in surgical patients, Thacker et al. [10] showed that a high volume of fluids administered on the day of surgery correlates significantly with longer hospital stay *(OR 1.10–1.40)* and higher overall cost *(OR 1.10–1.50)*. These authors also pointed out that, despite the implementation of clinical practice guidelines, knowledge of fluid therapy management among physicians is still deficient. This observation was echoed by Cordero et al. [11] who observed that more than 40% of specialists consulted believed there was a need for more education in fluid therapy, particular regarding indication, leading the authors to conclude that specific training programs, guidelines, and consensus fluid management protocols are needed.

The aim of this observational, transversal, multicentre study was to obtain current data on fluid therapy management by anaesthesiologists in Spain during the perioperative period in scheduled and urgent surgery in adult patients. We also analysed the infused fluid type, volume administered and monitorization used.

## Material and methods

### Design

Observational, transversal, multicentre study. Patients were included on 2 alternate days (18 and 20 February 2019) in all participating hospitals, with a follow-up period of up to 24 hours from each patient’s inclusion. In the case of patients who underwent outpatient surgery, follow-up continued until hospital discharge.

This study was approved by the ethics committee of Bellvitge University Hospital - Barcelona, with approval number HTF-FLU-2018-01 and other Hospitals (Annex 1) The patient provided written consent. All methods were carried out in accordance with the Spanish Agency for Drugs and Health Products (AEMPS): SED-HEA- 2018-0 and it was carried out according to the Declaration of Helsinki. It was registered in Clinical Trials: NCT03630744.

### Study population

We included patients over 18 years of age undergoing elective or emergency surgery over a period of 24 hours in the two study days. The exclusion criteria were interventions performed with local anaesthesia outside the surgical area, surgeries that did not require the presence of an anaesthesiologist, and ophthalmological surgery.

In order to classify and group patients by risk, comorbidity and surgery type we used the adapted *Risk Stratification Before Elective Surgery *(https://www.uclahealth.org/anes/risk-stratification) (Annex 2), stratifying patients in low- and high-risk and surgical procedures as low-risk, intermediate-risk, and high- or very high-risk.

The patients were classified according to the following distribution: Surgery Low-Intermediate and Patient Low-risk, Surgery Low-Intermediate and Patient High-risk, Surgery High-Very high and Patient Low-risk, Surgery High-Very high and Patient High-risk.

### Study variables

Data was recorded at each centre using an individual electronic case registration form designed specifically for the Fluid Day study.

For each patient we recorded demographic data, comorbidity data according to the Helixhauser classification [12], and surgery data. We also recorded the volume of fluid administered (operating room, post-anaesthesia care unit [PACU] and critical care unit) for up to 24 hours following the inclusion period (defined as the natural interval from the beginning of the surgical intervention up to 24 hours later), except in the case of outpatients, where follow-up ended with the patient's discharge.

The type of fluids administered were grouped as: crystalloids: Normal Saline 0.9%, Ringer Lactate, Isofundin®, Plasmalyte®, Glucose Serum 5%, Glucose serum 10 %, Glucosaline serum, and colloids: Hydroxyethyl starch 130/0.4 (HEA), Gelatine, Albumin 5%, Albumin 20%. The total volume administered during the intra and postoperative period and the total volume were recorded in millilitres (mL).

To adjust the total volume administered per patient, we added all the fluids, adding to the total volume of excipients for any drug (expressing the results in millilitres per kilogram – ml/Kg- and millilitres per kilogram per hour - ml/Kg/h) of surgery. The contribution of the volume of excipients to the total volume was also calculated as a percentage (%).

Monitoring used during the perioperative period was analysed and defined as: Non-invasive monitoring (Non-invasive blood pressure (NIBP): Electrocardiogram (EKG), pulsoximetry SpO2) , and Invasive haemodynamic monitoring if at least one of the following strategies was included: Invasive blood pressure (IBP), central venous pressure (CVP), pulmonary artery catheter (PAC)

cardiac output (CO), pulmonary thermodilution (PT), transpulmonary lithium dilution (TLD), transoesophageal echocardiography (TOE), systolic volume variation (SVV), systolic pressure variation (SPV), pulse pressure variation (PPV), plethysmography variation index (PVI).

We identified patients receiving an extra contribution of volume guided or not by protocol.

The number of patients who received any blood component (%) in each group of patients was included in the registry as a variable related to blood loss.

Study data were collected and managed using REDCap electronic data capture tools hosted at IDIBELL version 8.11.9. REDCap (Research Electronic Data Capture). The database was closed on 5 May 2019**.**

### Statistical analysis

This is an exploratory study to describe fluid therapy practice and management in Spain. All tertiary level Spanish hospitals were invited to participate. The expected response rate, above 60%, yielded a potential sample size of more than 3,500 patients, as all operations had to be included in the study. This was sufficient to achieve a precision of 3% or greater under a scenario of maximum variability p = q = 0.5. As this study has no primary hypothesis, the significance level from that analysis is not a reliable indicator, so no p-values are presented in tables [13].

The number of cases and percentages are presented as categorical variables; continuous variables are shown as mean and standard deviation (SD), or median and interquartile rank, depending on whether data distribution was normal. The normality of variables was assessed with graphs (QQ-Plot, density and standard deviations). Variables were presented for all surgeries and stratified by type of surgery and type of patient. Types of crystalloids used were presented by type of surgery and patient risk in graphics with percentages and 95% confidence interval calculated with exact binomial. Crystalloid Volume (ml/kg) was presented in a density plot separated by study groups. Analyses were performed with R software version 3.6.1 (2019-07-05*)* [14].

## Results

### Study population

**Fig. 1:**
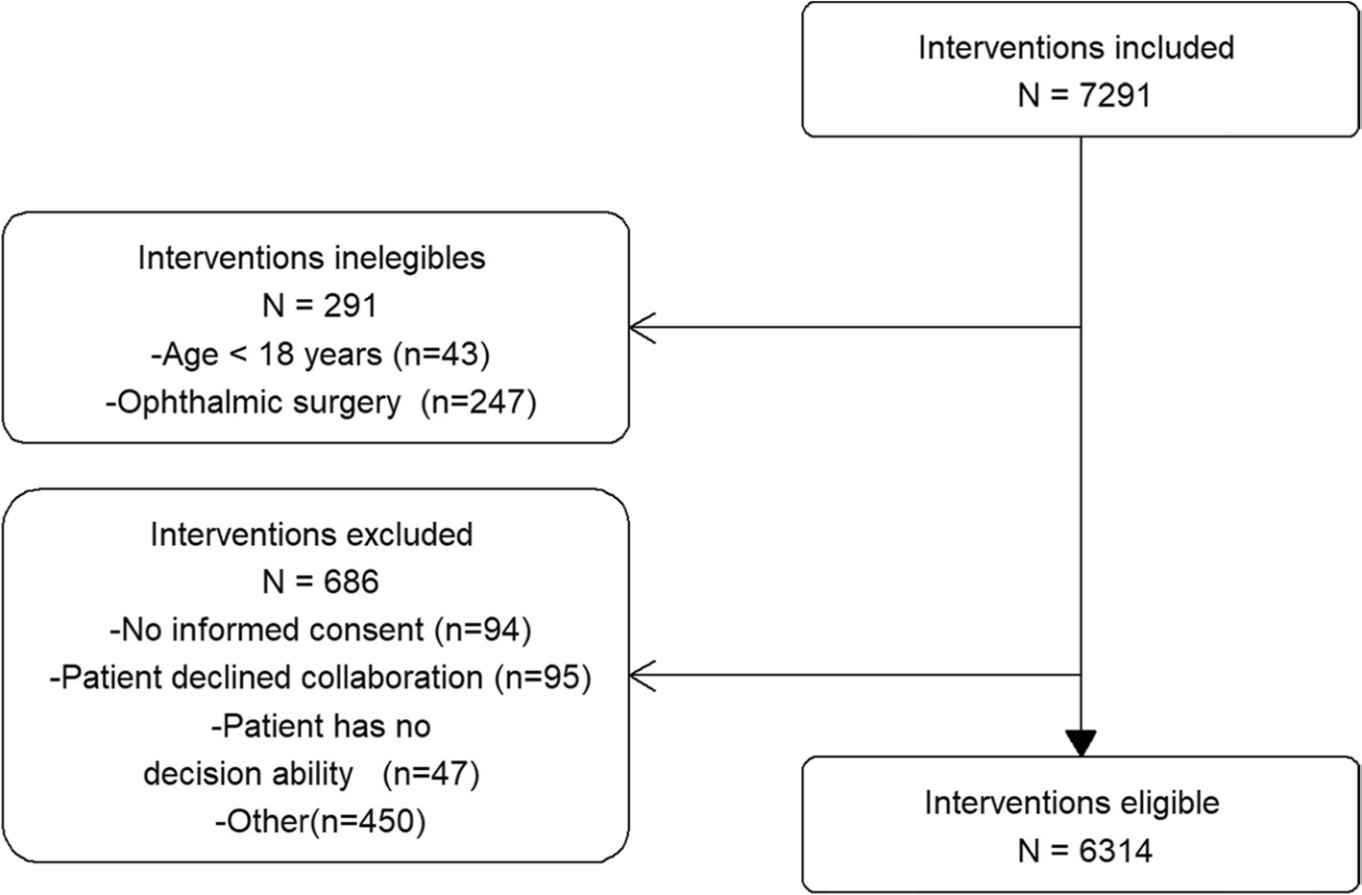
Flowchart

A total of 7291 patients participated in the study, 291 were excluded for not meeting the inclusion criteria, and 686 were excluded for other reasons, the most frequent being incomplete records (450 cases) (Fig. 1). A total of 6,314 patients from 131 different hospitals were included for analysis.

Of the total number of patients analysed, 3.223 (50.1%) were women, the mean age (SD) of the participants was 57.8 (17.1) years with BMI tending towards overweight (mean [SD]: 28.0 34.0) (Table 1). In total, 4669 patients (74.4%) had some associated comorbidity, and we observed a median [Q1;Q3] of 4.00 [2.00; 5.00] comorbidities in the high-risk group, hypertension being the most common comorbidity at 2411 patients (39.0%) in all subgroups (Table 1). Most high-risk surgery patients (485 [87.5%]) were ASA III.

**Table 1:** Demographic characteristics and clinical profile of patients

	ALL	Low-intermediate risk surgery & low risk patient	Low-intermediate risk surgery & high risk patient	High-very high risk surgery & low risk patient	High-very high risk surgery & high risk patient	N
	*N=6314*	*N=3830*	*N=943*	*N=987*	*N=554*	
**Sex**, N (%)						6314
♂ N (%)	3091 (49.0%)	1719 (44.9%)	537 (56.9%)	472 (47.8%)	363 (65.5%)	
♀ N (%)	3223 (51.0%)	2111 (55.1%)	406 (43.1%)	515 (52.2%)	191 (34.5%)	
Years, Mean (SD)	57.8 (17.1)	52.4 (16.4)	70.5 (13.7)	59.9 (15.0)	69.1 (11.9)	6314
**BMI** Mean (SD)	28.0 (34.0)	27.8 (43.4)	29.1 (6.86)	27.7 (4.93)	28.7 (6.05)	6200
Comorbidity, N (%)	4669 (74.4%)	2447 (64.3%)	924 (98.5%)	760 (77.6%)	538 (97.5%)	6274
Number of comorbidities^a^, Median [Q1;Q3]	2.00 [1.00;3.00]	1.00 [1.00;2.00]	3.00 [2.00;4.00]	2.00 [1.00;2.00]	4.00 [2.00;5.00]	4578
Hypertension^b^, N (%)	2411 (39.0%)	959 (25.7%)	659 (70.9%)	382 (39.5%)	411 (74.9%)	6183
Obesity^b^, N (%)	1859 (30.1%)	960 (25.7%)	370 (39.8%)	314 (32.4%)	215 (39.2%)	6183
Diabetes, N (%)	913 (14.8%)	298 (7.98%)	295 (31.7%)	117 (12.1%)	203 (37.0%)	6183
COPD^b^, N (%)	655 (10.6%)	251 (6.72%)	201 (21.6%)	81 (8.37%)	122 (22.2%)	6183
Depression^b^, N (%)	492 (7.96%)	276 (7.39%)	98 (10.5%)	77 (7.95%)	41 (7.47%)	6183
Hypothyroidism^b^, N (%)	468 (7.57%)	276 (7.39%)	88 (9.46%)	67 (6.92%)	37 (6.74%)	6183
Other neurological diseases^b^, N (%)	430 (6.95%)	143 (3.83%)	177 (19.0%)	38 (3.93%)	72 (13.1%)	6183
Solid tumour without metastasis^b^, N (%)	427 (6.91%)	162 (4.34%)	110 (11.8%)	72 (7.44%)	83 (15.1%)	6183
Cardiac arrhythmia^b^, N (%)	425 (6.87%)	77 (2.06%)	205 (22.0%)	30 (3.10%)	113 (20.6%)	6183
Peripheral vascular disease^b^, N (%)	371 (6.00%)	69 (1.85%)	131 (14.1%)	29 (3.00%)	142 (25.9%)	6183
Elixhauser score, Median [Q1;Q3]	1.00 [0.00;2.00]	1.00 [0.00;1.00]	3.00 [2.00;4.00]	1.00 [0.00;2.00]	3.00 [2.00;4.00]	6314
**ASA class**, N (%)						6314
I	1100 (17.4%)	955 (24.9%)	0 (0.00%)	145 (14.7%)	0 (0.00%)	
II	3717 (58.9%)	2875 (75.1%)	0 (0.00%)	842 (85.3%)	0 (0.00%)	
III	1355 (21.5%)	0 (0.00%)	870 (92.3%)	0 (0.00%)	485 (87.5%)	
IV-V^c^	142 (2.25%)	0 (0.00%)	73 (7.74%)	0 (0.00%)	69 (12.5%)	

*ASA* American Society of Anaesthesiology, *BMI* body mass index, *COPD* chronic obstructive pulmonary disease

^a^Patients with comorbidities

^b^Ten comorbidities more prevalent

^c^One patient had ASA V

Most surgeries were scheduled (5.692 [91.7%]). The most frequent type of surgery was orthopaedic (1795 [28.4%]); 243 patients (43.9 %) in the group of high - risk surgical patients underwent cardiac, vascular and thoracic surgery, and 324 patients (34.4%) considered high surgical risk underwent general surgery and low-risk digestive low surgery (Table 2).

**Table 2:** Surgical characteristics of the study population

	ALL	Low-intermediate risk surgery & low risk patient	Low-intermediate risk surgery & high risk patient	High-very high risk surgery & low risk patient	High-very high risk surgery & high risk patient
	*N=6314*	*N=3830*	*N=943*	*N=987*	*N=554*
**Duration (min)**, Median [Q1;Q3]	80.0 [50.0;120]	65.0 [45.0;110]	87.0 [50.0;135]	100 [65.0;154]	130 [87.0;220]
**Type**, N (%)					
Scheduled	5692 (91.7%)	3453 (91.3%)	795 (85.8%)	939 (97.9%)	505 (93.9%)
Urgent	512 (8.25%)	327 (8.65%)	132 (14.2%)	20 (2.09%)	33 (6.13%)
**Type**, N (%)					
Orthopaedic & traumatology	1795 (28.4%)	1032 (26.9%)	254 (26.9%)	372 (37.7%)	137 (24.7%)
General surgery	1633 (25.9%)	1182 (30.9%)	324 (34.4%)	75 (7.60%)	52 (9.39%)
Maxillofacial, plastic & ENT	777 (12.3%)	626 (16.3%)	72 (7.64%)	54 (5.47%)	25 (4.51%)
Urology	771 (12.2%)	410 (10.7%)	179 (19.0%)	133 (13.5%)	49 (8.84%)
Gynaecology	605 (9.58%)	484 (12.6%)	53 (5.62%)	63 (6.38%)	5 (0.90%)
Cardiac, thoracic & vascular	502 (7.95%)	28 (0.73%)	34 (3.61%)	197 (20.0%)	243 (43.9%)
Neurosurgery	172 (2.72%)	68 (1.78%)	27 (2.86%)	51 (5.17%)	26 (4.69%)
Other	59 (0.93%)	0 (0.00%)	0 (0.00%)	42 (4.26%)	17 (3.07%)
**Destination**, N (%)					
PACU	4906 (78.1%)	3215 (84.4%)	707 (75.3%)	689 (69.9%)	295 (53.6%)
Critical care unit	1305 (20.72%)	547 (14.35%)	223 (23.78%)	286 (29.03%)	249 (45.32%)
Ward	74 (1.18%)	49 (1.29%)	9 (0.96%)	10 (1.02%)	6 (1.09%)

*PACU* post-anaesthesia care unit

The postoperative follow-up of 4906 patients (78.1%) was performed in the post-anaesthesia care unit (PACU); 239 high-risk patients (43.5%) undergoing high-risk surgeries were followed up in critical care units (Table 2).

### Type of fluid administered

**Fig. 2:**
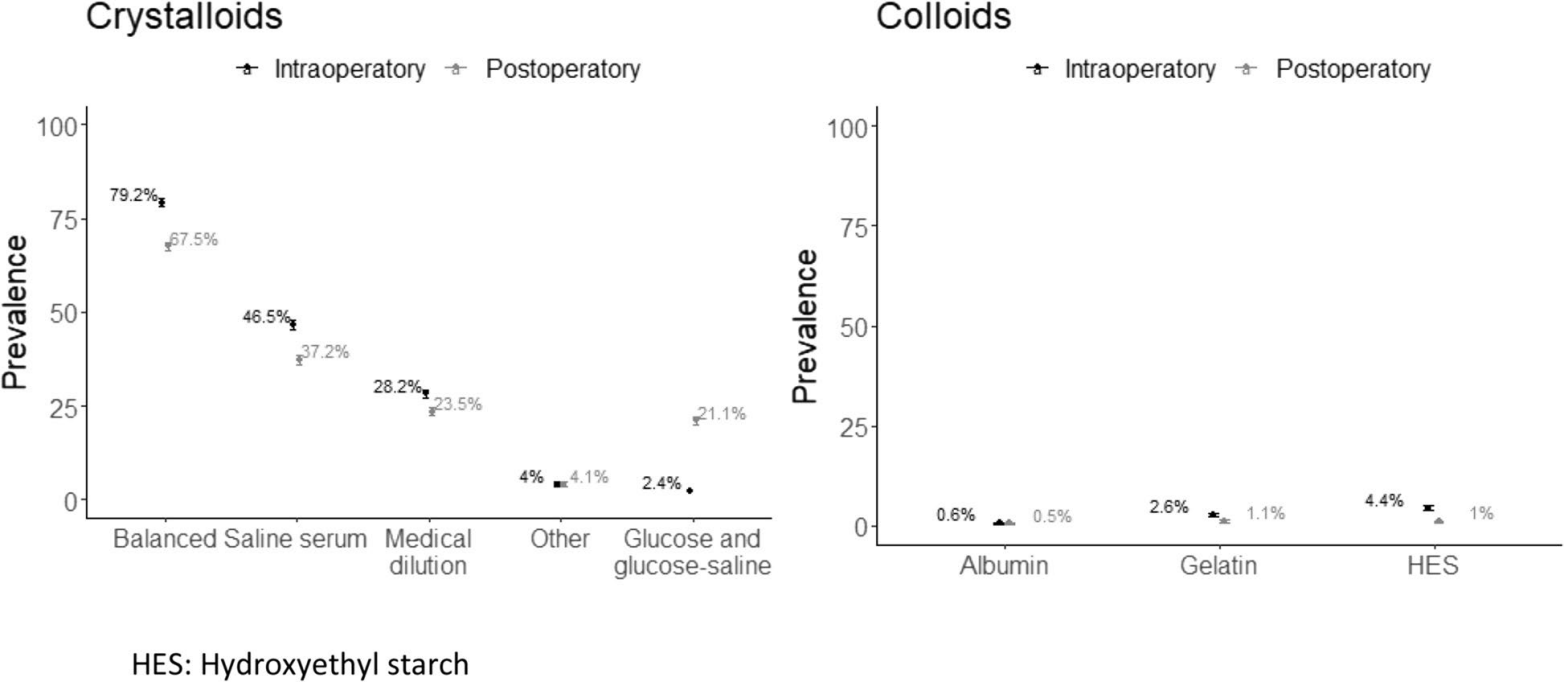
Fluid type (Prevalence and IC[95%]). HES: Hydroxyethyl starch

The most widely used intraoperative fluids were balanced crystalloids in 4912 patients (79.2% of the total volume of fluid), and normal saline 0.9% in 2883 patients (46.5% of the total volume of fluid). In the first 24 postoperative hours, balanced crystalloids were used in in 3825 (67.5%) patients, and saline in 2109 patients (37.2%), also as the percentage of the total volume of fluid Fig. 2, Table 3. A single crystalloid was used in 25% of surgeries, and 2 types of crystalloids were used in 50% of surgeries. In the other cases, other combinations of fluids were used; 21.1% of patients also received glucose solutions in the first 24 postoperative hours (Table 3).

**Table 3:** Type of fluids administered in the intraoperative period and the first 24 postoperative hours

	ALL	Low-intermediate risk surgery & low risk patient	Low-intermediate risk surgery & high risk patient	High-very high risk surgery & low risk patient	High-very high risk surgery & high risk patient	N
	*N=6203*	*N=3758*	*N=924*	*N=975*	*N=546*	
Type of fluids administered in the intraoperative period						
Crystalloids						
Crystalloids, N (%)	6203 (100%)	3758 (100%)	924 (100%)	975 (100%)	546 (100%)	6203
Number of Crystalloids, Median [Q1;Q3]	2.00 [1.00;2.00]	2.00 [1.00;2.00]	2.00 [1.00;2.00]	2.00 [1.00;2.00]	2.00 [1.00;2.00]	6203
Balanced, N (%)	4912 (79.2%)	2973 (79.1%)	699 (75.6%)	802 (82.3%)	438 (80.2%)	6203
Saline, N (%)	2883 (46.5%)	1651 (43.9%)	462 (50.0%)	475 (48.7%)	295 (54.0%)	6203
Excipients, N (%)	1749 (28.2%)	1019 (27.1%)	269 (29.1%)	297 (30.5%)	164 (30.0%)	6203
Other, N (%)	250 (4.03%)	146 (3.89%)	42 (4.55%)	42 (4.31%)	20 (3.66%)	6203
Glucose & glucose-saline, N (%)	150 (2.42%)	51 (1.36%)	47 (5.09%)	15 (1.54%)	37 (6.78%)	6203
Colloids						
Colloids, N (%)	474 (7.51%)	153 (4.06%)	104 (11.3%)	120 (12.3%)	89 (16.4%)	6207
Number of Colloids, Median [Q1;Q3]	1.00 [1.00;1.00]	1.00 [1.00;1.00]	1.00 [1.00;1.00]	1.00 [1.00;1.00]	1.00 [1.00;1.00]	474
Starch, N (%)	274 (4.41%)	93 (2.47%)	60 (6.49%)	79 (8.13%)	42 (7.75%)	6207
Gelatine, N (%)	163 (2.63%)	52 (1.38%)	36 (3.90%)	37 (3.81%)	38 (7.01%)	6207
Albumin, N (%)	37 (0.60%)	9 (0.24%)	12 (1.30%)	5 (0.51%)	11 (2.03%)	6207
Type of fluids administered in the first 24 postoperative hours						
Crystalloids						
Crystalloids, N (%)	5668 (100%)	3467 (100%)	848 (100%)	897 (100%)	456 (100%)	5668
Number of Crystalloids, Median [Q1;Q3]	1.00 [1.00;2.00]	1.00 [1.00;2.00]	1.00 [1.00;2.00]	1.00 [1.00;2.00]	1.00 [1.00;2.00]	6314
Balanced, N (%)	3825 (67.5%)	2370 (68.4%)	554 (65.3%)	613 (68.3%)	288 (63.2%)	5668
Normal saline, N (%)	2109 (37.2%)	1237 (35.7%)	318 (37.5%)	360 (40.1%)	194 (42.5%)	5668
Excipients, N (%)	1331 (23.5%)	740 (21.3%)	217 (25.6%)	226 (25.2%)	148 (32.5%)	5668
Other, N (%)	232 (4.09%)	132 (3.81%)	30 (3.54%)	41 (4.57%)	29 (6.36%)	5668
Glucose and glucose-saline, N (%)	1195 (21.1%)	565 (16.3%)	229 (27.0%)	235 (26.2%)	166 (36.4%)	5668
Colloids						
Colloids, N (%)	144 (2.45%)	43 (1.19%)	25 (2.87%)	41 (4.47%)	35 (7.54%)	5879
Number of colloids, Median [Q1;Q3]	1.00 [1.00;1.00]	1.00 [1.00;1.00]	1.00 [1.00;1.00]	1.00 [1.00;1.00]	1.00 [1.00;1.00]	144
Starch, N (%)	57 (0.97%)	22 (0.61%)	9 (1.03%)	14 (1.53%)	12 (2.59%)	5879
Gelatine, N (%)	64 (1.09%)	15 (0.41%)	14 (1.61%)	21 (2.29%)	14 (3.02%)	5879
Albumin, N (%)	31 (0.53%)	6 (0.17%)	6 (0.69%)	7 (0.76%)	12 (2.59%)	5879

Colloids were used intraoperatively in 466 patients (7.5%) and in 75% or more of the surgeries a single type was used (Median [Q1;Q3]: 1.00 [1.00; 1.00]). The most frequent combination was HEA (274 (4.41%)) and gelatine (163 (2.63%). In the first 24 postoperative hours, at least 1 colloid was administered in 144 patients (7.51%) (Fig. 2, Table 3). HEA was the most widely used intraoperative colloid (274 [4.41%]) and gelatine was the most widely used in the first 24 postoperative hours (64 [1.09%]). Albumin, a natural colloid, was used in the same proportion in both periods (37 [0.60%] and 31 [0.53%] patients, respectively), without observing differences between groups (Fig. 2, Table 3). High-risk patients undergoing high-risk surgery were given greater volume of colloids in both periods (89 [16.4%] intra-operative and 35 [7.54%] first 24 postoperative hours) (Table 3).

### Volume of fluid administered

In the intraoperative period, a median volume [Q1;Q3] of crystalloids administered vs volume per hour of surgery (ml/Kg/h) was 8.29 ml/Kg [5.56;12.3] vs 6.67 ml/Kg/h [3.83; 8.17]) + administration medium in low-risk patients undergoing low/intermediate risk surgery; 9.17 [5.81; 14.1] vs 6 ml/Kg/h [4.04; 9.08] in high-risk patients undergoing low/intermediate risk surgery; 12.0 [7.78; 18.3] vs 6.41 ml/Kg/h [4.36; 9.33] in low-risk patients undergoing high-risk surgery; and 13.2 [7.62; 8.21] vs 5.46 ml/Kg/h [3.83; 8.17] in high-risk patients undergoing high-risk surgery (Table 4).

**Table 4:** Overall fluids administered in the intraoperative period and the first 24 postoperative hours

	ALL	Low-intermediate risk surgery & low risk patient	Low-intermediate risk surgery & high risk patient	High-very high risk surgery & low risk patient	High-very high risk surgery & high risk patient	N
	*N*=6158	*N*=3729	*N*=917	*N*=971	*N*=541	
Total volume administered in the intraoperative period						
Total ml/Kg Median [Q1;Q3]	8.33 [5.43;13.3]	7.69 [5.00;11.8]	8.33 [5.26;13.3]	11.1 [6.91;17.2]	12.5 [6.86;20.6]	6142
Total ml/Kg/h surgery, Median [Q1;Q3]	6.35 [4.17;9.52]	6.67 [4.23;10.0]	6.00 [4.04;9.08]	6.41 [4.36;9.33]	5.46 [3.83;8.17]	6039
Total ml/Kg + excipients, Median [Q1;Q3]	9.04 [6.00;14.3]	8.29 [5.56;12.3]	9.17 [5.81;14.1]	12.0 [7.78;18.3]	13.2 [7.62;21.8]	6158
% excipients, N (%)						6142
0%-3%	4457 (72.6%)	2728 (73.3%)	658 (72.2%)	678 (69.9%)	393 (72.9%)	
3%-6%	19 (0.31%)	9 (0.24%)	4 (0.44%)	5 (0.52%)	1 (0.19%)	
>6%	1666 (27.1%)	985 (26.5%)	249 (27.3%)	287 (29.6%)	145 (26.9%)	
**Total volume administered in the** first 24 postoperative hours						
Total ml/Kg Median [Q1;Q3]	5.26 [2.86;9.24]	4.62 [2.67;7.65]	5.56 [2.94;10.0]	6.90 [3.85;12.8]	8.73 [4.15;18.3]	5511
Total ml/Kg/h surgery, Median [Q1;Q3]	3.92 [2.15;7.32]	3.90 [2.12;7.19]	3.96 [2.14;7.88]	4.01 [2.35;7.10]	3.88 [2.00;7.50]	5420
Total ml/Kg + excipients, Median [Q1;Q3]	5.56 [3.06;9.80]	5.00 [2.78;8.20]	5.92 [3.11;10.6]	7.40 [4.16;13.9]	9.21 [4.44;20.8]	5607
% excipients, N (%)						5392
0%-3%	4269 (79.2%)	2666 (81.1%)	618 (76.9%)	670 (77.9%)	315 (71.6%)	
3%-6%	5 (0.09%)	2 (0.06%)	3 (0.37%)	0 (0.00%)	0 (0.00%)	
>6%	1118 (20.7%)	620 (18.9%)	183 (22.8%)	190 (22.1%)	125 (28.4%)	
**Overall volume of fluid administered (intra- and** first 24 postoperative hours**)**						
Total ml/Kg Median [Q1;Q3]	14.5 [9.45;22.6]	13.0 [8.61;19.3]	14.9 [9.38;23.2]	18.9 [11.9;30.5]	21.4 [12.8;37.0]	5495
Total ml/Kg/h surgery, Median [Q1;Q3]	10.9 [7.27;16.7]	11.1 [7.30;17.2]	10.7 [7.20;16.8]	10.9 [7.63;16.2]	10.2 [6.99;14.7]	5406
Total ml/Kg + excipients, Median [Q1;Q3]	15.4 [10.3;24.1]	13.9 [9.52;20.5]	15.7 [10.4;24.5]	20.2 [13.3;32.4]	22.7 [14.1;40.9]	5596
% excipients, N (%)						5455
0%-3%	3514 (64.4%)	2190 (65.9%)	510 (62.7%)	544 (62.4%)	270 (60.7%)	
3%-6%	65 (1.19%)	30 (0.90%)	12 (1.47%)	10 (1.15%)	13 (2.92%)	
>6%	1876 (34.4%)	1104 (33.2%)	292 (35.9%)	318 (36.5%)	162 (36.4%)	

In first 24 postoperative hours, the median [Q1;Q 3] ml/Kg crystalloids plus 5.00 excipients administered was 3.90 [2.78; 8.20], 5.92 [3.11; 10.6], 7.40 [4.16; 13.9], and 9.21 [4.44; 20.8], respectively (Table 4). Total Median [Q1; Q3] ml/Kg crystalloids administered was 13.9 [9.52; 20.5], 15.7 [10.4; 24.5], 20.2 [13.3; 32.4] and 22.7 [14.1; 40.9], respectively.

**Fig. 3:**
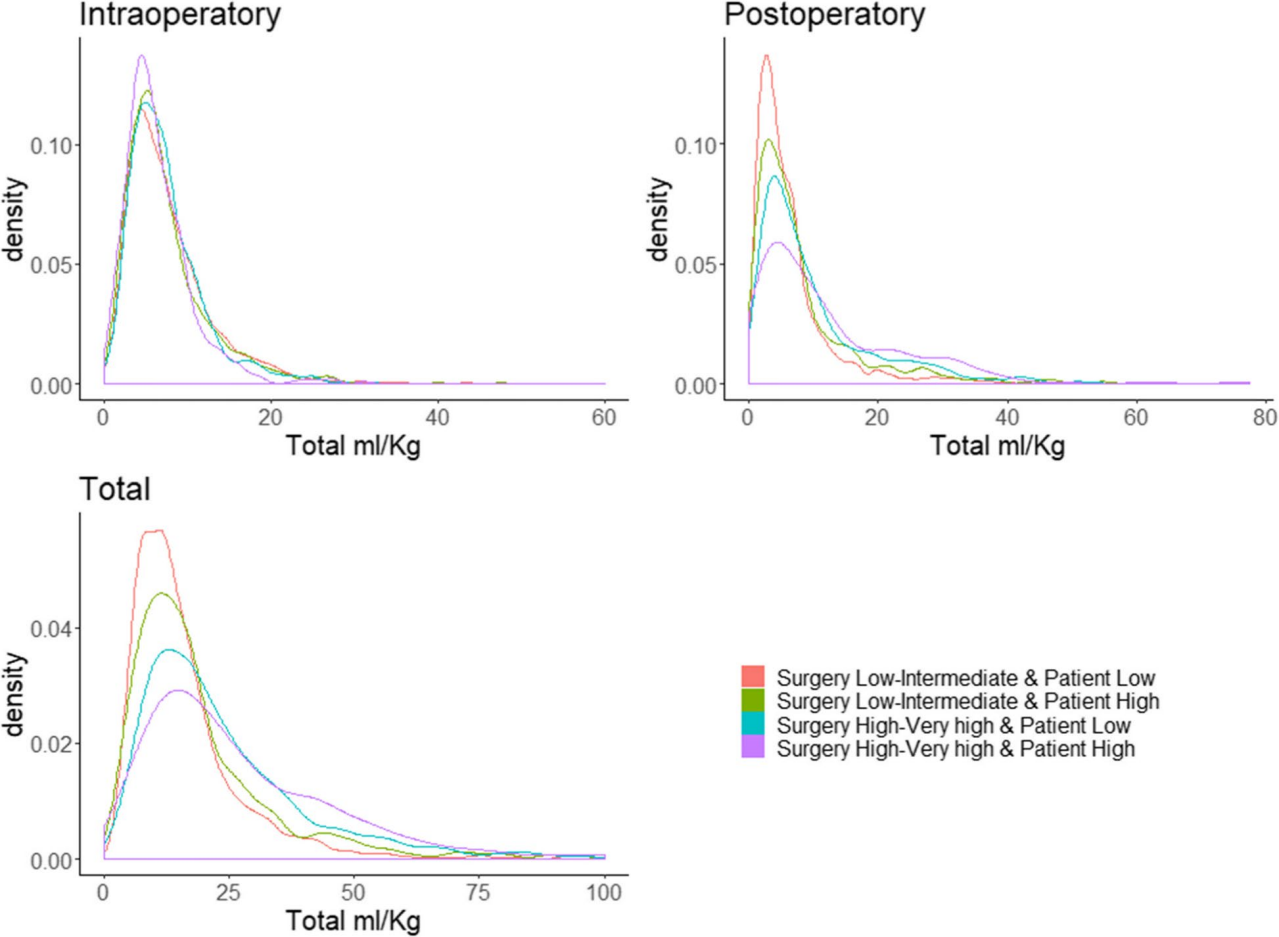
Density plot of ml/Kg of Crystalloids. * 15 patients with more than 100 ml/Kg in total were removed. In the densities graph we observe that, during the intraoperative period in most of the surgeries, the total ml/kg are concentrated between 0 and 20. In contrast, in the postoperative period we see that, the greater the patient and surgery risk, the more variability in the total ml/kg administered. This same variability is observed in the total administered volume in both the intra and postoperative periods

Figure 3 shows that the highest density of patients received between 0 and approximately 20 ml/Kg of crystalloids during the intraoperative period, being distributed equally between groups. During the first 24 postoperative hours, we see that in low-risk patients the highest patient density continues to be from 0 to 20 ml/Kg, while when the risk of the patient and of the surgery increases, the density is dispersed, exceeding 20 ml/Kg. The group with the highest risk is the one with the lowest density, between 0 and 20 ml/Kg.

In the overall volume of crystalloids administered, we see that the density between groups differs, the higher the risk of the patient and the type of surgery, the higher the density of patients between 0 and 25 ml/Kg.

In 3514 patients (64.4%), the volume added as drug excipients represented 3% or less of the total volume of crystalloid administered, between 3% and 6% in 65 (1.19%) patients, and more than 6% in 1876 (34.4%), with no differences according to groups (Table 4).

### Fluid therapy management: monitoring used and protocol

Non-invasive monitoring was used in 99.1% of patients (Table 5). Regarding the use of invasive hemodynamic monitoring, we included invasive BP in 206 patients (38%) in the high surgical risk group, and CVP in 101 (18%) patients in the same group (Table 5). Other types of monitoring considered to be invasive were used in a greater proportion in the group of 85 (15%) high surgical risk patients (Table 5).

**Table 5:** Fluid therapy management and monitoring strategy

	ALL	Low-intermediate risk surgery & low risk patient	Low-intermediate risk surgery & high risk patient	High-very high risk surgery & low risk patient	High-very high risk surgery & high risk patient	N
	*N=6246*	*N=3791*	*N=928*	*N=980*	*N=547*	
**Monitoring**						
**Non-invasive monitoring** (NIBP, EGC, SpO2), N (%)	6187 (99.1%)	3757 (99.1%)	917 (98.8%)	968 (98.8%)	545 (99.6%)	6246
IBP, N (%)	630 (10.1%)	133 (3.52%)	122 (13.2%)	169 (17.3%)	206 (38.0%)	6215
CVP, N (%)	243 (3.91%)	40 (1.06%)	41 (4.43%)	61 (6.27%)	101 (18.6%)	6207
Diuresis control, N (%)	1915 (30.9%)	830 (22.0%)	357 (38.6%)	415 (42.7%)	313 (57.4%)	6207
Advanced haemodynamic monitoring, N (%)	206 (3.32%)	32 (0.85%)	36 (3.90%)	53 (5.42%)	85 (15.6%)	6213
**Fluid therapy management in the intraoperative period**						
Patients receiving additional fluids, N (%)						6173
No	5778 (93.6%)	3593 (96.0%)	828 (90.4%)	894 (92.1%)	463 (85.3%)	
By protocol	93 (1.51%)	14 (0.37%)	20 (2.18%)	22 (2.27%)	37 (6.81%)	
Without protocol	302 (4.89%)	136 (3.63%)	68 (7.42%)	55 (5.66%)	43 (7.92%)	
**Fluid therapy management in the** first 24 postoperative hours						
Patients receiving additional fluids, N (%)						5865
No	5729 (97.7%)	3580 (98.8%)	841 (97.3%)	884 (96.9%)	424 (90.8%)	
By protocol	32 (0.55%)	3 (0.08%)	6 (0.69%)	5 (0.55%)	18 (3.85%)	
Without protocol	104 (1.77%)	39 (1.08%)	17 (1.97%)	23 (2.52%)	25 (5.35%)	

*CVP* Central venous pressure, *ECG* Electrocardiogram, *NIBP* Non-invasive blood pressure, *SpO2* oxygen saturation, *IBP* Invasive Blood Pressure

No extra volume contributions were recorded in 93.6 % of patients in the intraoperative period and 97.7% in the first 24 postoperative hours. The recorded volume input episodes were differentiated according to the existence or absence of a specific protocol (Table 5). Per protocol volume administration was performed mainly in the high-risk surgical group in 37 patients (6.81%) out of a total of 547 patients who formed this subgroup in the intraoperative period, and in 18 patients (3.85%) in the first 24 postoperative hours. The volume administered in the absence of a protocol was similar in both periods and for the same group of patients - 43 (7.92%) in the intraoperative period and 25 (5.35% in the first 24 postoperative hours (Table 5).

## Discussion

Our Fluid Day study shows that crystalloids are the main intravenous fluid used in the perioperative period, and that balanced solutions are used more often for this purpose, while normal saline is still frequently administered to surgical patients. We know that there are differences between these types of crystalloid solutions, and that this has generated controversy regarding their management in the surgical patient. Firstly, 0.9% chloride-rich saline causes a higher degree of acidosis and dose-dependent hyperchloremia, which can favour smooth muscle vascular contraction that can reduce renal perfusion [15–17]. In a study carried out in healthy volunteers who received 2 litres of normal saline 0.9% vs. balanced crystalloid - Plasma- Lyte ®, perfusion in the renal artery and total urine output significantly decreased, with an increase in extravascular fluid compared to Plasma- Lyte ® [18]. These findings support the notion that hyperchloremia can reduce renal cortical perfusion [19]. Similarly, a large observational study showed that the use of Plasma - Lyte ® versus normal saline 0.9% in patients undergoing major abdominal surgery produced less acute kidney failure and need for renal replacement therapy [20].

Two recent studies also compared the administration of balanced crystalloids and normal saline 0.9% in critical and non-critical patients [16, 17]. Both studies showed a lower incidence of acute kidney damage with balanced solutions, and a lower incidence of death and new-onset renal replacement therapy in critically ill patients. However, the SOLAR study [21] showed no clinically significant differences in postoperative complications with Ringer lactate ® (balanced crystalloid) or normal saline 0.9% in elective orthopaedic surgery patients and colorectal cancer patients [21].

Despite this, prior to conducting the Fluid Day study, normal saline 0.9% was not recommended during major surgery [22], since its administration was associated with hyperchloremia, metabolic acidosis and acute renal injury in the postoperative period [5, 23–25]. However, normal saline 0.9% was used in 45% of patients included in the Fluid Day study, specifically in 50% of high-risk patients and high-risk surgeries.

Moreover, the Fluid Day study has shown that increased patient and/or surgery risk was associated with an increase in the total amount of fluid administered within 24 hours, while the total volume/kg/hr administered in the intraoperative period was lower in patients with high risk, and patients with high anaesthesia and surgical risk received the lowest volume. The optimal amount of perioperative maintenance fluid is a highly controversial issue, and this inevitably leads to variability in its administration and total volume contribution [3, 8], although in recent years volume overload avoidance has been recommended, as it increases postoperative complications [1, 9, 26–28]. Recently, a cohort study conducted in 500 US hospitals in adult patients undergoing colorectal surgery and primary hip or knee arthroplasty [9] found a significant association between liberal fluid administration and worse outcomes (increased cost and total hospital stay), as well as increased presence of postoperative ileus, especially in patients undergoing colorectal surgery. Interestingly, the authors also found that restrictive fluid administration (25% lower volume administered with respect to the liberal approach) was also associated with worse outcomes, particularly acute kidney injury in high-risk patients undergoing high-risk surgery [9, 29].

In general, the literature suggests that fluid management during the perioperative period should be based on algorithms and protocols, because they provide better outcomes, especially in terms of volume or total amount administered [8, 9, 13, 26]. Currently, restrictive fluid maintenance therapy is recommended for enhanced recovery after surgery pathways [7, 30]. The RELIEF study [9] showed that the restrictive approach led to a median of 1.7 litres of intraoperatively fluid administered compared to 3 L with the liberal approach [9]. Patients in the restrictive group had proportionally greater acute renal injury than patients in the liberal group (8.6% vs. 5%, p <0.001). These authors recommended a fluid system to provide a positive fluid balance of 10 to 12 ml/Kg/h during major abdominal surgery, and 1.5 ml/Kg/h in the first 24 h postoperative hours.

In the Fluid Day study, we administered around 6.35 ml/Kg ^of^ volume in any group during the intraoperative period, and 3-4 ml/Kg in the first 24 postoperative hours, affirming the aforementioned hypothesis. However, overall fluid administration including the intraoperative period and the first 24 postoperative hours, as well as the administration of fluid as drug excipients, placing the total balance at 15.4 ml/Kg.

Other studies [31] used a crystalloid maintenance infusion of 10 ml/Kg/h, while the OPTIMISE study [32] administered 1 ml/Kg/h crystalloid as maintenance fluid. Both studies were performed in the UK and in similar surgeries. Therefore, variability in dosage persists.

Other types of surgery not associated with significant losses do not usually require high intraoperative administration of fluids to achieve a moderate positive fluid balance at the end of surgery, for example, in low-risk patients undergoing low / intermediate risk surgery. For this reason, the total volume administered in our study was 13ml/Kg [9.52; 20.5]. For these patients, a maintenance balanced crystalloid fluid ratio of 1 to 3 ml/Kg/h would be recommended as an early transition to oral fluid therapy after surgery [22].

Regarding colloid administration, 7.3% of patients received at least one colloid during surgery (mostly HEA), while 2.2% received some type of colloid in the first 24 postoperative hours (mostly gelatine). The mean volume administered was 500 mL. High-risk patients and those who underwent high-risk surgery received colloids in a higher percentage. These results appear to be consistent with the clinical context and show that the administration of colloids was performed in a restricted manner during the study period. Clinical studies comparing colloid and crystalloid administration for goal-directed volume replacement in major abdominal surgery showed that the latter did not reduce the serious complications or hospital stay, but also did not cause long-term or acute renal toxicity [33]. The recent Fluid Loading in Abdominal Surgery: Saline vs Hydroxyethyl Starch (FLASH) trial [31] compared administration of hydroxyethyl-starch (HEA) vs unbalanced solution in 775 patients at high risk of postoperative renal injury in major abdominal surgery. HEA administration was associated with lower fluid balance, better hemodynamic parameters, and lower vasopressors during surgery; however, they found no differences in their composite outcome of mortality and postoperative complications or postoperative AKI [34].

In our study, 24.4% of patients (1541) underwent high-risk surgery, 23.7% were high-risk patients (1497) and only 8.7% (554) were high-risk patients undergoing high-risk surgeries. Very few (19%) were admitted to a critical care unit in the postoperative period, giving an idea of ​​the risk of liberal or restrictive fluid therapy in this group. Despite recommendations [35], only 15% of patients undergoing high-risk surgeries included in the Fluid Day study underwent invasive haemodynamic monitoring, and less than 10% received goal-directed therapy. A small proportion of patients received fluid according to a goal-directed strategy, such as fluid challenge with monitoring strategy (per protocol) or fluid load without monitoring strategy. The use of goal-directed fluid therapy is gradually being incorporated into clinical practice for high-risk patients undergoing high-risk surgery [8, 36]. Cannesson et al. [37] studied the impact of the systematic implementation of a goal-directed perioperative hemodynamic strategy in patients undergoing high-risk abdominal surgery, finding an 18% decrease in the length of hospital stay and a significant decrease in postoperative complications from 39% to 25%.

In a recent meta-analysis including 45 randomized controlled trials (*N* = 6344 participants), Sun et al. [38] reported that a goal-directed therapy was associated with a significant reduction in short- and long-term mortality in patients undergoing major abdominal surgery.

The Fluid Day study is the first observational study on the management of perioperative fluid therapy carried out in different Spanish hospitals. It provides a general description of routine clinical practice of anaesthesiologists in terms of fluid management in patients with different surgical and anaesthesia risks.

The variability shown in Fluid Day highlights the need to improve the management of perioperative fluid therapy. The forthcoming publication of the ideal fluid pattern according to anaesthetic and surgical risk, together with the spread of this knowledge and its implementation and subsequent follow-up will show whether the Fluid Day study has benefited patients.

### Limitations

First, this is an observational study with a minimum follow-up time of 24 hours, a limitation that has not allowed us to analyse safety aspects in the short and long term. This cross-sectional study, unfortunately, did not collect clinical or patient-reported outcomes.

There was also no comparison with a standard of care, because the main objective of the study was to observe the clinical management of perioperative fluid therapy with no intention of making comparisons with an established standard practice.

We did not associate the variability observed in our study with any postoperative outcome. While this is of interest in future studies, we believe that the existence of such variability is in itself an important issue to address to improve the quality of fluid therapy management.

### Conclusions

The results of this cross-sectional observational study suggest that balanced solutions are the most widely used crystalloids in any type of surgery and type of patient in our surgical setting. Fluid management is performed without the use of monitoring or goal-directed protocols in most patients and surgeries, and in a small proportion of high-risk patients undergoing high-risk surgeries. This suggests that advanced monitoring is only valued in high-risk patients undergoing high-risk surgeries but does not specifically target fluid therapy management.

We believe that the results obtained in this study are important because they show that there is currently great variability in clinical practice and in the management of perioperative fluid therapy. It appears that more seriously ill patients tend to receive less fluids but often with little advanced monitoring.

## Supplementary Information

**Additional file 1.**

**Additional file 2.**

**Additional file 3.**

**Additional file 4.**

## Data Availability

The datasets used and/or analysed during this study are available from the corresponding author on reasonable request.

## Abbreviations

CEIC: Clinical Practice Ethics Committee
PACU: Post-anaesthesia care unit
HEA: Hydroxyethyl starch 130/0.4
mL: Millilitres
ml/Kg: Millilitres per kilogram
ml/Kg/h: Millilitres per kilogram per hour
NIBP: Non- invasive blood pressure
EKG: Electrocardiogram
SpO2: Pulsoximetry
IBP: Invasive blood pressure
CVP: Central venous pressure
PAC: Pulmonary artery catheter
CO: Cardiac output
PT: Pulmonary thermodilution
TLD: Transpulmonary lithium dilution
TOE: Transoesophageal echocardiography
SVV: Systolic volume variation
SPV: Systolic pressure variation
PPV: Pulse pressure variation
PVI: Plethysmograph variation index
SD: Standard deviation
BMI: Body mass index

## References

[1] Lechat JP, Van der Linden P (2019). Fluid therapy in the intraoperative setting. Transfus Apher Sci.

[2] Rehm M, Hulde N, Kammerer T, Meidert AS, Hofmann-Kiefer K (2019). State of the art in fluid and volume therapy: a user-friendly staged concept. Anesthesist..

[3] Malbrain MLNG, Langer T, Annane D, Gattinoni L, Elbers P, Hahn RG, De Laet I, Minini A, Wong A, Ince C (2020). Intravenous fluid therapy in the perioperative and critical care setting: executive summary of the International Fluid Academy (IFA). Ann Intensive Care.

[4] Malbrain MLNG, Jacobs R, Perner A (2019). The search for the holy grail continues: the difficult journey towards the ideal fluid!. J Crit Care.

[5] Miller TE, Myles PS (2019). Perioperative fluid therapy for major surgery. Anesthesiology..

[6] Messina A, Robba C, Calabrò L, Zambelli D, Iannuzzi F, Molinari E, Scarano S, Battaglini D, Baggiani M, De Mattei G (2021). Association between perioperative fluid administration and postoperative outcomes: a 20-year systematic review and a meta-analysis of randomized goal-directed trials in major visceral/noncardiac surgery. Crit Care.

[7] Calvo-Vecino JM, Ripollés-Melchor J, Mythen MG, Casans-Francés R, Balik A, Artacho JP, Martínez-Hurtado E, Serrano Romero A, Fernández Pérez C, Asuero de Lis S (2018). Effect of goal-directed haemodynamic therapy on postoperative complications in low – moderate risk surgical patients: a multicenter randomized controlled trial (FEDORA trial). Br J Anaesth.

[8] Della Rocca G, Vetrugno L, Tripi G, Deana C, Barbariol F, Pompeiet L (2014). Liberal or restricted fluid administration: are we ready for a proposal of a restricted intraoperative approach?. BMC Anesthesiol.

[9] Myles PS, Bellomo R, Corcoran T, Forbes A, Peyton P, Story D, Christophi C, Leslie K, McGuinness S, Parke R (2018). Restrictive versus liberal fluid therapy for major abdominal surgery. N Engl J Med.

[10] Thacker JKM, Mountford WK, Ernst FR, Krukas MR, Mythen MMG (2016). Perioperative fluid utilization variability and association with outcomes: considerations for enhanced recovery efforts in sample US surgical populations. Ann Surg.

[11] Cordero MA, Moreno JM, Gomis P, Valero MA, Calleja MA (2012). Pilot study of intravenous fluid therapy management in adult patients at a tertiary care hospital. Nutr Hosp.

[12] Elixhauser A, Steiner C, Harris DR, Coffey RM (1998). Comorbidity measures for use with administrative data. Med Care.

[13] Harrington D, D'Agostino RB, Gatsonis C, Hogan JW, Hunter DJ, Normand ST, Drazen JM, Hamel BM (2019). New guidelines for statistical reporting in the journal. N Engl J Med.

[14] Team. RC. A language and environment for statistical computing. In: R Core Team (2018) R: A Language and Environment for Statistical Computing. R Foundation for Statistical Computing, Vienna. https://www.R-project.org; 2017.

[15] Hansen PB, Jensen BL, Skøtt O (1998). Chloride regulates afferent arteriolar contraction in response to depolarization. Hypertension..

[16] Self WH, Semler MW, Wanderer JP, Wanderer JP, Wang L, Byrne DW, Collins SP, Slovis CM, Lindsell CJ, Ehrenfeld JM (2018). Balanced crystalloids versus saline in noncritically Ill adults. N Engl J Med.

[17] Semler MW, Self WH, Wanderer JP, Ehrenfeld JM, Wang L, Byrne DW, Stollings JL, Kumar AB, Hughes CG, Hernandez A (2018). Balanced crystalloids versus saline in critically Ill adults. N Engl J Med.

[18] Pfortmueller CA, Fleischmann E (2016). Acetate-buffered crystalloid fluids: current knowledge, a systematic review. J Crit Care.

[19] Potura E, Lindner G, Biesenbach P, Funk GC, Reiterer C, Kabon B, Schwarz C, Druml W, Fleischmannet E (2015). An acetate-buffered balanced crystalloid versus 0.9% saline in patients with end-stage renal disease undergoing cadaveric renal transplantation: a prospective randomized controlled trial. Anesth Analg.

[20] Shaw AD, Bagshaw SM, Goldstein SL, Scherer LA, Duan M, Schermer CR, Kellum JA (2012). Major complications, mortality, and resource utilization after open abdominal surgery: 0.9% saline compared to plasmalyte. Ann Surg.

[21] Maheshwari K, Turan A, Makarova N, Ma C, Sakr Esa WA, Ruetzler K, Barsoum S, Kuhel AG, Ritchey MR, Higuera-Rueda C (2020). Saline versus lactated ringer's solution: The saline or Lactated Ringer's (SOLAR) trial. Anesthesiology..

[22] Ripollés-Melchor J, Chappell D, Espinosa A, Mhyten MG, Abad-Gurumeta A, Bergese SD, Casans-Francés R, Calvo-Vecino JM (2017). Perioperative fluid therapy recommendations for major abdominal surgery. Via RICA recommendations revisited. Part I: Physiological background. Rev Esp Anestesiol Reanim.

[23] Burdett E, Dushianthan A, Bennett-Guerrero E, Cro S, Gan TJ, Grocott MPV, et al. Perioperative buffered versus non-buffered fluid administration for surgery in adults. Cochrane Database Syst Rev. 2012;12. 10.1002/14651858.CD004089.pub2 23235602

[24] Saw MM, Chandler B, Ho KM (2012). Benefits and risks of using gelatin solution as a plasma expander for perioperative and critically ill patients: a meta-analysis. Anaesth Intensive Care.

[25] Orbegozo Cortés D, Rayo Bonor A, Vincent JL (2014). Isotonic crystalloid solutions: a structured review of the literature. Br J Anaesth.

[26] Soni N (2009). British Consensus Guidelines on Intravenous Fluid Therapy for Adult Surgical Patients (GIFTASUP): Cassandra’s view. Anaesthesia.

[27] Clinical N, Center G. Intravenous fluid therapy in adults in hospital. Commissioned by the National Institute for Health and Care Excellence 2013. https://pubmed.ncbi.nlm.nih.gov/25340240/.

[28] Brandstrup B, Tønnesen H, Beier-Holgersen R, Hjortsø E, Ørding H, Lindorff-Larsen K, Rasmussen MS, Lanng C, Wallin L, Iversen LH (2003). Effects of intravenous fluid restriction on postoperative complications: comparison of two perioperative fluid regimens - a randomized assessor-blinded multicenter trial. Ann Surg.

[29] Shin CH, Long DR, McLean D, Grabitz SD, Ladha K, Timm FP, Thevathasan T, Pieretti A, Ferrone C, Hoeft A (2018). Effects of Intraoperative Fluid Management on Postoperative Outcomes: A Hospital Registry Study. Ann Surg.

[30] Cecconi M, Fasano N, Langiano N, Divella M, Costa MG, Rhodes A, Della RG (2011). Goal-directed haemodynamic therapy during elective total hip arthroplasty under regional anesthesia. Crit Care.

[31] Challand C, Struthers R, Sneyd JR, Erasmus PD, Mellor N, Hosie KB, Minto G (2012). Randomized controlled trial of intraoperative goal-directed fluid therapy in aerobically fit and unfit patients having major colorectal surgery. Br J Anaesth.

[32] Pearse RM, Harrison DA, MacDonald N, Gillies MA, Blunt M, Ackland G, Grocott MPW, Ahern A, Griggs K, Scott R (2014). Effect of a perioperative, cardiac output-guided hemodynamic therapy algorithm on outcomes following major gastrointestinal surgery a randomized clinical trial and systematic review. JAMA..

[33] Kabon B, Sessler DI, Kurz A, Crystalloid–Colloid Study Team. Effect of Intraoperative Goal-directed Balanced Crystalloid versus Colloid Administration on Major Postoperative Morbidity: A Randomized Trial. Anesthesiology. 2019; 130(5): 728-744. 10.1097/ALN.0000000000002601 30882476

[34] Futier E, Garot M, Godet T, Biais M, Verzilli D, Ouattara A, Huet O, Lescot T, Lebuffe G, Dewitte A (2020). Effect of hydroxyethyl starch vs saline for volume replacement therapy on death or postoperative complications among high-risk patients undergoing major abdominal surgery: The FLASH Randomized Clinical Trial. JAMA..

[35] Vincent JL, Pelosi P, Pearse R, Payen D, Perel A, Hoeft A, Romagnoli S, Ranieri VM, Ichai C, Forget P (2015). Perioperative cardiovascular monitoring of high-risk patients: a consensus of 12. Crit Care.

[36] Huette P, Abou Arab O, Beyls C, Mahjoub Y (2019). Fluid challenge: de la théorie à la pratique. Anesth Renim.

[37] Cannesson M, Ramsingh D, Rinehart J, Demirjian A, Vu T, Vakharia S, Imagawa D, Yu Z, Greenfield S, Kainet Z (2015). Perioperative goal-directed therapy and postoperative outcomes in patients undergoing high-risk abdominal surgery: a historical-prospective, comparative effectiveness study. Crit Care.

[38] Sun Y, Chai F, Pan C, Romeiser JL, Gan TJ (2017). Effect of perioperative goal-directed hemodynamic therapy on postoperative recovery following major abdominal surgery-a systematic review and meta-analysis of randomized controlled trials. Crit Care.

