# Supplementary material for: Observational study on fluid therapy management in surgical adult patients
Source: BMC Anesthesiol. 2021 Dec 13;21:316. doi: 10.1186/s12871-021-01518-z (PMC8667365; doi:10.1186/s12871-021-01518-z)
Supplement: Supplementary file 1 — Additional file 1. [file 12871_2021_1518_MOESM1_ESM.docx]

| Hospital Name - City - |
| --- |
| Fundació Puigvert. Barcelona |
| Hospital Universitario La Moraleja, Sanitas - Madrid |
| Hospital Clínic de Barcelona |
| Hospital Marina Baixa de la Vila Joiosa - Alicante |
| Hospital Universitario de Torrejón-Torrejón de Ardoz - Madrid |
| Hospital Universitario Sagrat Cor de Barcelona |
| HOSPITAL ÁLVARO CUNQUEIRO- COMPLEJO HOSPITALARIO UNIVERSITARIO DE VIGO |
| Hospital Universitario 12 de Octubre - Madrid |
| Hospital de Alta Resolución de Guadix (Granada) |
| Hospital de Rehabilitación y Traumatología Virgen del Rocío. Sevilla |
| Complexo Hospitalario de Ourense |
| Complejo hospitalario universitario de Santiago de Compostela |
| Hospital Clínico San Carlos . Madrid |
| Quiron Salud Torrevieja |
| Hospital Universitario Ramón y Cajal, Madrid |
| HOSPITAL CAN MISSES - IBIZA |
| Hospital Ntr. Sra. del Prado- Talavera de la Reina (Toledo) |
| Hospital Povisa - VIGO |
| Hospital Universitario de Fuenlabrada |
| Hospital Verge dels Lliris - Alcoi |
| CONSORCIO SANITARIO ANOIA ( HOSPITAL DE IGUALADA ) |
| Hospital La Plana - Vilarreal - Castellón |
| Hospital Universitario Fundación Alcorcón- Madrid |
| Hospital General de La Palma |
| HOSPITAL UNIVERSITARIO DE MÓSTOLES |
| H.G.U. Gregorio Marañón- Madrid |
| Hospital General Universitario Reina Sofía- Murcia |
| Clinica universidad de navarra |
| Hospital Universitari Santa Maria - Lleida |
| Hospital General de Castellón |
| Hospital Universitario San Agustin de Aviles |
| Complejo Hospitalario Universitario de Albacete |
| Hospital Universitario San Juan de Alicante - San Juan de Alicante |
| Hospital Universitari St. Joan- Reus |
| Complejo Asistencial de Zamora, Hospital Virgen de la Concha, Zamora |
| Complejo Hospitalario Universitario de Pontevedra |
| Hospital de Galdakao-Usánsolo. Bizkaia |
| COMPLEJO HOSPITALARIO DE SEGOVIA |
| Hospital del Mar - Barcelona |
| Hospital de Jerez de la Frontera |
| Hospital Universitario Marques de Valdecilla. Santander |
| Hospital General de Granollers - Barcelona |
| Hospital de la Ribera. Alzira. Valencia |
| Hospital Verge de la Cinta de Tortosa |
| Hospital Universitari Joan XXIII Tarragona |
| Clínica IMQ Zorrotzaurre - Bilbao |
| Hospital Donostia – san Sebastián |
| Hospital General Universitario Valencia |
| Hospital Son llatzer-Palma de Mallorca |
| Hospital Fremap Majadahonda. Madrid |
| Althaia Xarxa Assistencial Universitària de Manresa - Manresa |
| Hospital Medina del Campo.valladolid |
| Hospital Central de la Cruz Roja San José y Santa Adela - Madrid |
| Hospital Universitario Río Hortega. Valladolid |
| HOSPITAL UNIVERSITARIO NTRA. SRA DE CANDELARIA (SANTA CRUZ DE TENERIFE) |
| Hospital Universitario de Canarias |
| Hospital Universitari General de Catalunya - Sant Cugat del Vallés |
| HOSPITAL UNIVERSITARIO VIRGEN MACARENA. SEVILLA |
| Hospital Clínico Universitario de Valladolid |
| Hospital Francesç de Borja de Gandía |
| Hospital Universitario Álava |
| Hospital de Zumárraga, OSI Goierri Alto Urola |
| Hospital General Universitario José María Morales Meseguer- Murcia |
| Hospital Sant Joan Despí Moisès Broggi |
| Hospital General de l'Hospitalet |
| Centre d'Atenció Integral Dos de Maig |
| Hospital General Universitario Elche |
| Hospital de Sagunto-Sagunto |
| HOSPITAL UNIVERSITARIO DOCTOR PESET, VALENCIA |
| Hospital universitario quironsalud Madrid |
| Hospital Juan Ramón Jiménez - Huelva |
| Hospital Lluis Alcanyís de Xàtiva |
| Complejo Hospitalario Universitario Insular Materno Infantil |
| Hospital Universitario de GRan Canaria Dr. Negrín-Las Palmas de Gran Canaria |
| Hospital Universitario La Paz |
| Hospital Universitario "Reina Sofía". Córdoba |
| HOSPITAL CLINICO UNIVERSITARIO DE VALENCIA |
| Doctor Jose Molina Orosa. Arrecife , Lanzarote |
| Hospital Ruber Internacional-Madrid |
| Hospital Universitario Guadalajara |
| Complejo Hospitalario Universitario A Coruña |
| CLINICA UNIVERSITARIA NAVARRA MADRID. |
| HOSPITAL CENTRAL DE LA DEFENSA GÓMEZ ULLA - MADRID |
| Complejo Hospitalario Universitario de Badajoz-Badajoz |
| Hospital Parc Tauli.Sabadell |
| Complejo Asistencial Universitario de León |
| Hospital Santa Creu i Sant Pau |
| HOSPITAL UNIVERSITARIO DE LA PRINCESA. |
| Hospital General Universitario de Alicante |
| Hospital de Mérida |
| Hospital Universitario Puerta de Hierro Majadahonda |
| Consorci Sanitari del Maresme, Hospital de Mataro- Mataro |
| Hospital Universitari Mútua Terrassa |
| ARNAU DE VILANOVA-LLIRIA. VALENCIA |
| Parc Sanitari Sant Joan de Déu, Sant Boi de Llobregat |
| Hospital Don Benito -Badajoz |
| Hospital Universitario Severo Ochoa (Leganés, Madrid) |
| Hospital Universitari i Politècnic La Fe |
| Hospital Universitari Bellvitge - L´Hospitalet |
| Hospital Universitario Cruces |
| HOSPITAL UNIVERSITARIO PRÍNCIPE DE ASTURIAS - ALCALÁ DE HENARES (MADRID) |
| Hospital Universitario Infanta Leonor - Madrid |
| Fundación Jiménez Díaz- Madrid |
| HOSPITAL GENERAL UNIVERSITARIO VIRGEN DE LA SALUD.ELDA. ALICANTE |
| Hospital Rey Juan Carlos - Mostoles |
| Hospital Vall d´Hebron - Barcelona |
| Hospital Universitario Virgen de la Arrixaca. El Palmar. Murcia |
| Hospital Universitario Miguel Servet. Zaragoza |
| Hospital Mancha Centro-Alcazar de San Juan _Ciudad Real |
| Hospital Reina Sofía Tudela |
| CH Torrecárdenas-Almería |
| Hospital Obispo Polanco, Teruel |
| Hospital Universitario Virgen de la Victoria de Málaga |
| Hospital Plató- Barcelona |
| Fundació Hospital Sant Joan de Déu, Martorell |
| Complejo hospitalario de Jaén |
| Hospital Virgen de la Salud - Toledo |
| HOSPITAL CLINICO UNIVERSITARIO LOZANO BLESA |
| Hospital Universitario Arnau de Vilanova _Lleida |
| Hospital Universitari Josep Trueta - Girona |
| Hospital Mutua Accidentes de Zaragoza - Zaragoza |
| Hospital universitario del Vinalopó |
| Fundación Hospital Espíritu Santo, Barcelona |
| Hospital de Alcañiz |
| Complejo Asistencial Universitario de Salamanca (CAUSA) |
| Complejo Hospitalario Universitario Insular Materno Infantil |
| Hospital QuironSalud Bizkaia |
| Hospital Universitario de Valme. Sevilla |
| Hospital Regional Universitario de Málaga |
| Complejo Hospitalario Universitario de Pontevedra |
