# Supplementary material for: Observational study on fluid therapy management in surgical adult patients
Source: BMC Anesthesiol. 2021 Dec 13;21:316. doi: 10.1186/s12871-021-01518-z (PMC8667365; doi:10.1186/s12871-021-01518-z)
Supplement: Supplementary file 2 — Additional file 2. [file 12871_2021_1518_MOESM2_ESM.docx]

Annex 1

Risk Stratification Before Elective Surgery (adapted) *(https://www.uclahealth.org/anes/risk-stratification*)

### Surgical risk stratification

| **Surgical risk** | **Types of surgery** |
| --- | --- |
| Low – Intermediate Risk | **Procedures associated with minimal physiologic effect and /or moderate changes in hemodynamics, risk of blood loss**   - Hernia repair - ENT procedures without planned flap or neck dissection - Diagnostic cardiac catheterization - Interventional radiology and endoscopy with stent placement - Cystoscopy - Intracranial and spine surgery - Gynaecologic and urologic surgery - Intra-abdominal surgery without bowel resection - Intra-thoracic surgery without lung resection - Cardiac catheterization procedures including electrophysiology studies, ablations, AICD, pacemaker |
| High – Very High Risk | **Procedures with possible significant effect on hemodynamics, blood loss and /or major impact on hemodynamics, fluid shifts, possible major blood loss**   - Colorectal surgery with bowel resection - Kidney Transplant - Major joint replacement (shoulder, knee, and hip) - Open radical prostatectomy, cystectomy - Major oncologic general surgery or gynaecologic surgery - Major oncologic head and neck surgery - Aortic surgery - Cardiac Surgery - Intra-thoracic procedures with lung resection - Major transplant surgery (heart, lung, liver) |

###

### Patient Medical Risk Stratification

Pre-existing medical problems confer risk for perioperative complications, including cardiac decompensation, respiratory failure, acute kidney injury, and postoperative delirium or cognitive dysfunction.

| **Patient risk** | **Patient’s commorbidity** |
| --- | --- |
| Low risk | - No known medical problems - Hypertension - Hyperlipidaemia - Asthma - Other chronic, stable medical condition without significant functional impairment - Age 70 or older - Non-insulin dependent diabetes - History of treated, stable CAD - Morbid obesity (BMI> 30) - Anaemia (haemoglobin <10) - Mild renal insufficiency |
|  |  |
| High risk | - Recent coronary stent - Chronic CHF - Insulin-dependent diabetes mellitus - Renal insufficiency: creatinine> 2 - Moderate COPD: FEV1 50% to 70% - Obstructive sleep apnoea - History of stroke or TIA - Known diagnosis of dementia - Chronic pain syndrome - Unstable or severe cardiac disease - Severe COPD: FEV1 <50% predicted - Use oxygen at home - Pulmonary hypertension - Severe liver disease - Severe frailty; physical incapacitation |
|  |  |
