# Supplementary material for: Observational study on fluid therapy management in surgical adult patients
Source: BMC Anesthesiol. 2021 Dec 13;21:316. doi: 10.1186/s12871-021-01518-z (PMC8667365; doi:10.1186/s12871-021-01518-z)
Supplement: Supplementary file 3 — Additional file 3. [file 12871_2021_1518_MOESM3_ESM.docx]

**Appendix 2**

**Hospitals and Researchers who participated in the Fluid Day study**

**Hospital Universitario Sagrat Cor de Barcelona**: Adriana Hervías Villar (Principal Investigator, PI), Astrid Álvarez Pérez, Federica Castelli, Nuria Sierra Medina, Esteban Díaz Jaimes, Susana Velásquez Jaimes, Laura Ruiz Villa, Jorge González Aguirre, María José Alvira Uribe, Espedito Brunetto, Ricard Valdés Arribas, Mireia Gili Bueno, Guido Muñoz Rojas, Maddalena Pasini, Carla Lorena Zenteno.

**Hospital Universitario 12 de Octubre - Madrid**: Raquel García Álvarez (PI), Francisco Pérez-Cerdá Silvestre, David Benguria Puebla, Rosalía Navarro Casado, Raquel Delestal Aldaria, Isabel de la Calle Gil, Pablo López-Arcas Calleja, Celia Méndez López del Hierro, Germán Sánchez Barbe, Clara Perosanz Silvo, Hugo Ahern Espinosa.

**Hospital Universitario Basurto- Bilbao:** M Pilar Rodrigo Casanova (PI), Luciano Aguilera Celorrio, Joseba González García, Alexander González Bada, Diana Ly Liu, Arantxa Echevarría Correas. Hospital de Alta Resolución de Guadix (Granada): Ignacio Velázquez Rivera (PI), Jorge Núñez Olea, Manuel Jesús Sánchez del Águila, Mª Ángeles Medina Cuesta, Carmen Melero Ramos, Elena Herrerías Ortiz, Pilar García Velasco.

**Clínica IMQ Zorrotzaurre - Bilbao:** Mª Begoña Girbau Campo (PI), Jorge Villalonga, María Elsa Ortega Sánchez, Julen Unzueta Serrano, Unai Valtierra Gómez, Borja García Casal, Miren Trujillo Onandia.

**Hospital de Rehabilitación y Traumatología Virgen del Rocío - Sevilla:** Rafael Rubio Romero (PI), Ignacio Jiménez López, Manuel Rico Borrego, Gabriel Yanes, Rosana Guerrero Domínguez, Ana Martínez Saniger.

**Hospital Universitari Santa Maria – Lleida:** Reis Drudis Morell (PI), Gregorio Marco Naya, Xènia Triquell Domeyo, Antonio Montero Matamala, Dolors del Pozo Garcia.

**Complexo Hospitalario de Ourense:** Leticia Gómez Viana (PI), Fermín Domínguez Hervella, Concepción Alonso González, Olalla Figueiredo González, Eva López López, Eva Villar Arcay, Marta Couñago Garrido, Nuria Carballo Loureiro, Raquel Ruido Dacal, Alberto Escobar Veiga.

**Complejo Hospitalario Universitario de Santiago de Compostela – A Coruña**: Laura Dos Santos Carregal (PI), Marta Carolina Freijeiro Gonzalez (PI), Julian Álvarez Escuredo, Manuel Taboada Muñiz, Maria del Mar Eiras Marino, Adrian Martinez Salgado, Sabela de Rio Fernandez,

**Hospital Marina Baixa de la Vila Joiosa - Alicante:** Alejandra Molines Cantó (PI), Francisco José Martínez Adsuar, Patricia Valls Linares, Anna Grabowska, Gracia barber ballester, Maria Isabel Tarí Bas, Cristina Munck Álvarez.

**Hospital Clínico San Carlos - Madrid:** Lourdes Durán (PI), Luis Santé, Dr. Enrique Garcia – Pelayo, Rubén Sanchez , Angela Muñoz de Solano, Brezo del Blanco, Laura Alvarez Mercadal, Ignacio Nuñez, Alexandra Alonso Morenza, Carlos Choza de Juan.

**Hospital Quironsalud Torrevieja - Alicante:** Ana Belén Tovar López (PI), Andrej Balik, Noelia Ruiz Vera, Julio Pedro Vinuesa Nuñez.

**Hospital Universitario Ramón y Cajal - Madrid:** Yolanda Diez Remesal (PI), David Pestaña, Pascual Crespo Aliseda, Hortensia Garcia de Quinto, Inés de la Hoz Polo, Lucia Pereira Torres, Marta Rámirez Gonzalez, Gerardo Arias Cuesta, Ana Mª López palacios, Berta Iglesias Gallego, Amal Azzam López, Javier Moya Moradas, Guillermo Bartolomé Rubio.

**Hospital Can Misses – Ibiza:** Jesús Alonso Cabello (PI), María Teresa Nogueiras Pérez, Diego Marín Guijarro, Sara Gallo Santacruz , Nerea Gómez Pérez.

**Hospital Nuestra Sra. del Prado- Talavera de la Reina - Toledo:** Pablo Gimeno Fernandez (PI), José Luis Gómez Agraz, Elzbieta Boze Stasiak-Pikula, Carla Iglesias Morales.

**Hospital Povisa - Vigo:** Paula Nespereira Garcia (PI), Rafael Cabadas, Dra. Mª Sonsoles Leal Ruiloba, Benigno Rodríguez Estévez, Dra. Marina Varela Rouco, Dra. Eva Rocha Peleteiro, Dr. Rafael Cabadas Avión,

**Hospital Universitario de Fuenlabrada - Madrid:** Jorge Almoguera Fernández (PI), José Olarra Nuel, Raquel Montoro Antón, Isabel Rodríguez Seguin, Lourdes Izquierdo Llanos, Andrea Cuadrado Mancy, Ana Zulema Castro Costoya, María Elena Civeira Marín, Noemí Rocío Pinto Sánchez, Ana María. Ana María Castillo Díaz, Luis Angel Bonilla Castillo, Maria Jose Guinaldo Elices, Juan del Arco Casas.

**Hospital La Plana - Vilarreal – Castellón:** Ana Nicolau Gozalbo (PI), Paloma Ripolles Martínez, Carmen Alcalde Sanchez, Lidia Vives Piqueras, Inmaculada Gimenez Jimenez, Francisca Montero Sanchez, Marian Daras Orenga, Montiel Redondo Castillo, Jose Pedro Calabuig Soler, Maria Angeles daras Orenga, Pilar Caro Gomez.

**Hospital Virgen de la Concha - Zamora:** Yaiza Beatriz Molero Diez (PI), Raquel Sanchis Dux (PI), Soledad Vega Cruz. Jesús Javier Cuello Azcárate, Francisco Antonio Ruiz Simón, Miguelina Eugenia Michel Tactuk, Víctor Javier Sánchez Hernando, José Luis González Rodríguez, Rebeca Martin Martin.

**Consorcio Sanitario Anoia - Hospital de Igualada – Barcelona:** Ana María Pedrero GIL (PI), Jose María Bausili Pons, Ana María Pedrero Gil , Joanna Grössl Melean.

**Hospital Universitario de Valme - Sevilla:** Tiburcio Vázquez Gutiérrez (PI), Dra Mercedes Echevarria Moreno, Pilar Gómez Reja, Reyes Morales Rodriguez, Raquel Ortiz de la Tabla, Lourdes Olmedo Granados, Inmaculada Sanchez Martin, Carlos Avila Zurita, Marta Reina Molina.

**Hospital Verge dels Lliris – Alcoi - Alicante:** José Luis Jover Pinillos (PI), Cristina Martinez Piera, José Pedro García Belmonte. Elodia Gregori Mahiques, Santiago Ruiz Casado, Reyes Pastor, Lorena Blanes, Mila Caldes, Javier Alonso.

**Hospital Universitario Fundación Alcorcón - Madrid:** Diana Zamudio Penko (PI), Santiago Garcia del Valle, Violeta María Heras Hernando, Pablo Redondo Martínez, Roberto Real Valdés, Natalia Gijón Herreros, Livia Gisbert de la Cuadra, Ana Kataryzna Grzanka, Laura Fernández Tellez, Patricia Gaite De Vicente, Patricia Navarro Echevarria.

**Hospital General de La Palma - Canarias:** Carolina Quiroz (PI), Rafael Bello Puentes, Dieter Weingärtner.

**Hospital Álvaro Cunqueiro- Complejo Hospitalario Universitario de Vigo**: Olga Martínez Chaves, Gerardo Baños Rodriguez, Pilar Aguirre Puig, Teresa Otero Amoedo, Elena Vilas Otero, Paz Prieto Requeijo, María de los Ángeles Orallo Morán, Denis Pereira Matalobos, Jesús García Rey, Verónica González Monzón, José Ramón Repáraz Andrade, Pedro Castellano Canda, Laura Alonso Prieto, Pedro Martínez Guitián, Kelly Johanna Millan Rodríguez, Gustavo Illodo Miramontes, María Jesús Fernández Cebrián, María Dolores González Sandoval, María Ángeles Gil Campelo, Silvia Picallo Vieito, Silvia González Chomón, Aser García Muñoz, Juan José Amate Pena, Adriana Rial Veloso, Fabián Barreiro Bouzón, Lorena Sánchez Oliva, Cora Díaz Aquino, Iria de la Torre Riveiro, Sara Sierra Cardalda, Carmen Llamas Pernas, Clara García Lorenzo, Gorka Ibarra Forneiro.

**Hospital General Universitario Santa Lucia – Cartagena - Murcia:** Olga Cecilia Correa Chacón (PI), Maria Elena Estelles Montesinos, Pablo Amate, Mario Eduardo Fagni, Miguel Sánchez Nicolas, Miguel Ángel Astrain Aguado.

**Hospital Universitario de Móstoles - Madrid:** Raquel Fernández-García, Rocío Ayala Soto, Francisco de Borja de la Quintana Gordon. Dra. Mª Visitación Álvarez García, Briseida Arrázola Cabrera, Lidia Mª Castro Freitas, Angela de Santos López, Ángel Callejo Martín, Elizabeth Miriam González González, Mario Inaudis Fajardo Pérez, Carmen Garrido Espa, Lara Guitiérrez García., Alicia Gutiérrez-Jodra Gamboa, Inmaculada Hidalgo Nuchera, Raquel Iglesias Blanco., Juan José Llavador Ros, Mercedes Martínez García, Begoña Menéndez Bodega, Mª Fe Muñoz Velázquez, Beatriz Isabel Nacarino Alcorta, Diana Marcela Narváez Cubillos, José Miguel Peñas Garrote, Isabel Mª Sepúlveda Gil, Yolanda Laporta Baez, Celddy Mireya Urquizo Torrico, Ricardo Iván Villamarín Tibadu, Rosa Zueras Batista, Lilian Valeria Bacuilima Brito, Diego García Simón, Carmen Portolés Díez, Margarita Merino Ruiz.

**Hospital General Universitario Gregorio Marañón- Madrid:** Rafael Ramos Fernández (PI), María Teresa López Gil, Pilar Benito Saz, Cristina Julia Lisbona Saez, Elena López Gil, Susana Díaz Ruano, Teresa del Castillo Fernández de Betoño, Irma María Barrio Pérez, María Lema Tomé, Alberto Calvo García, Pablo Tomás Aznar García, Luis Olmedilla Arnal, Jose Pérez Peña.

**Hospital General Universitario Reina Sofía- Murcia:** Dra. Carmen Mª López López (PI), Aída Blaya Solana, Mª Teresa Yepes García, Jose Miguel López López, Kateryna Mikhno, Rebeca González Celdrán.

**Clinica Universitaria de Navarra:** Francisco Hidalgo Martínez (PI), Alfredo Panadero Sánchez, Jorge Mendoza Sorrondegui, Iñigo Rubio Baines, Elena Méndez Martínez, Andrea Lara Jiménez.

**Hospital Clínic de Barcelona:** Nuria Martín Marata (PI), Graciela Martínez Pallí, Ramón Adàlia Bartolomé, Mercè Agustí Lasús, Amàlia Alcón Domínguez, M Teresa Anglada Casas, Maria José Arguis Giménez, Rosario Armand Ugon, Jaume Balust Vidal, Misericordia Basora Macaya, Isabel Belda Tortosa, Joan Josep Beltran Garcia, Annabel Blasi Ibáñez, Xavier Borrat Frígola, Angel Caballero del Pozo, Andrea Calvo Barrera, Albert Carramiñana Domínguez, Enrique Jesús Carrero Cardenal, Maria José Carretero Casado, Montserrat Celemín Miguel, Alberto Coy Serrano, Nicolás Gonzalo De Riva solla, María Pinar De Santos Maroto, Maria Elena Del Río Morales, M.Neus Fàbregas Julià, Carlos Ferrando Ortola, Guillermina Fita Rodríguez, Jaume Fontanals Dotras, Marta Garcia Orellana, Pedro Luís Gambús Cerrillo, Carmen Gomar Sancho, Isabel Gracia Sánchez, Josep Antoni Gràcia Solsona, Lidia Gómez López, Paola Hurtado Restrepo, Cristina Ibáñez Esteve, Irene León Carsi, Antonio López Hernández, Manuel López Baamonde, Marta Magaldi Mendaña, Julia Martínez Ocón, Purificación Matute Jiménez, Ricard Mellado Artigas, Jordi Mercadal Mercadal, Concepción Monsalve Maestro, Luis Alfonso Moreno Cuartas, Guido Andrés Muñoz Rojas, Ricard Navarro Ripoll, Antonio José Ojeda Niño, Miriam Fiore Panzeri, Juan Manuel Perdomo Linares, Ana María Plaza Moral, Roger Pujol Muncunill, Raquel Risco Martínez, Irene Rovira Canudas, M Carmen Roux Carmona, Ana Ruiz Pardos, Franscesc Xavier Sala Blanch, Fátima Salazar García, Josep Martí Sanahuja Blasco, Gerard Sánchez-Etayo Gianotti, Beatriz Tena Blanco, Francisco Javier Tercero Machín, Monterrat Tió Felip, Marta Ubré Lorenzo, Ricard Valero Castell, Marina Vendrell Jordà, Elisabeth Zavala Zegarra.

**Hospital General de Castellón:** Laura Jordá Sanz (PI), Miguel Vila Sánchez, Xavier Cabedo Vidal, Maria Gellida  Vilarroig, Aranzazu Ordóñez Arana, Olga Aznar Millán, Raquel Coscarón García, José Miguel España Pamplona, Jose C Beltrán Traver, Ana Belén Ramírez Sánchez, Javier Novella Peris, Marta Soriano Hervás, Tágara Fayos Alemany, Maria Isabel de Miguel Cabrera, Daniel Robles Hernández, Maria Jesús Arcusa Mon.

**Hospital Universitario San Agustin de Aviles:** Dr Juan Artamendi, Jesús Bujarrabal Martínez, Gabriel Mella Pérez, Beatriz Laserna Cocina, Pablo de la Rica Fernández, María Jesús González Miranda, Diana de la Uz Díaz, Laura Morante Carrión, Marta López Pérez, Lorena España Fuente, Elena Méndez Redondo, Angel Gómez Martín, Andrea Manzano Patallo, Cristina Rodríguez, Marina Rodríguez Rodríguez , Covadonga Gutiérrez González, Paz Alonso Pérez, Esther Ovies García, Virginia Chico Marcos, Sandra Fernández Sampedro, Maira Morillo González, Jorge Álvarez González , José Ignacio Fernández Fernández, Ana María Martínez Garcia, Sarai Santos Martin, Olga Beatriz Alvarez García, Marta Arias Díaz, Marta Blanco Collazos, Paula Menéndez Garcia, Manuela Garcia Gonzalez, Angeles Herrero Moreno, Rocio Pelaez Suarez, Patricia Sánchez Pascual, Mónica García Mayo, Paula Gonzalez Selgas, Ana María Pérez García, Maria Josefa Pérez Santiago, Paz Lopez Caliero, Ana Asensio Benito, Patricia Fernández Sánchez, Lucia Álvarez Prendes, Marta Heres Campa.

**Complejo Hospitalario Universitario de Albacete:** Pedro Manuel Canales Lara (PI), Julián de Capadocia Rosell, Laura Fernández Ruipérez.

**Hospital Universitario de Torrejón-Torrejón de Ardoz - Madrid:** Javier Alexander Salazar Duque (PI), Ludy Erika Calderon Barajas, Elena Garcia Fernandez.

**Hospital Universitario San Juan de Alicante:** Sergio Tejada Ortega (PI), Jorge Hernando Sáez, Javier Alejandro Agulló Agulló, Ezequiel García-Ripoll Catalan, Vicente Gilabert Gomis, Hipolito Pérez Moya, Salvadora Pérez Roig, Maria Del Carmen García De La Torre, Paula Arnau Papí, Vera Garcia García.

**Hospital Universitari St. Joan- Reus - Tarragona:** Pilar Sala Francino (PI), Guillem Bujosa Portells.

**Complejo Hospitalario Universitario de Pontevedra:** Marina Varela Durán, María de los Ángeles Carro Roibal, Pilar Díaz Parada, Marina Barreiro Torres, Susana Marcelo Brage, Cristina Barreiro Pardal, María Rodríguez Losada, Ángela González Vilar, Lucía Penide Villanueva, Manuel Formoso Fojo, Rafael Andreu.

**Complejo Hospitalario de Segovia:** Fco. Javier Garcia Miguel, Ana Isabel Diez Lobo, Angel Luis Fuentes de Frutos, Blanca Ortega, Raquel Gómez Sanz, Adriana Sofía Ramírez.

**Hospital de Galdakao-Usánsolo - Bizkaia:** Mikel Arzuaga Esquino (PI), Unai Ortega Mera, Olatz Arámburu Uriarte, Iraia Ereño Ealo, Eider García Pérez, Pilar Romero Rojano, Marta Errazquin Aguirre, Sofia Irigoyen Miro, Beatriz Del Val Villanueva, Beatriz Esnaola Iriarte, Alba Fernandez Fernandez De Quincoces, Maria San Juan Gonzalez, Elisa Diez Castillo, Claudia Velasco Oficialdegu, Nerea Azpiazu Landa, Zuriñe Lauzirikaauregui, Aitziber Etxebarria Agorria, Hector Miguel Alcalde, Saioa Riaño Onaindia.

**Hospital del Mar – Barcelona:** Beatriz Fort Pelay (PI), Fernando Escolano Villen, Angie Catherine Carpintero Cruz, Daniel Amorós Ruiz , Elvira Bisbe Vives, Pedro Rivera Soria, Maider Puyada Jauregui.

**Hospital de Jerez de la Frontera – Sevilla:** Jacobo José Fedriani de Matos (PI), Maria Milagrosa Santana Pineda, Eloy López Silva, Ana Martinez De Castro, Ana Moreno Martín, Juan Diego Leal Del Ojo, Maria Pérez García, Fatima García Herrera, Elena García Gómez, Ana Quintero Salvago, Maria Muñoz Zambrano, Marta Ruiz Mayo, Nadia Tuyani Soliman, Laura Barrios Rodríguez, Maria Delgado Moya.

**Hospital Universitario Marques de Valdecilla - Santander:** Jose Manuel Rabanal Llevot, Jose Luis Rabago Moriyon, Guillermo Tejon Perez, Eva Maria Capa Fuertes, Isabel M Ruiz Garcia, Daniel Garcia Barrigon, M Angelina Rodriguez Caballero, Rodrigo Sancho Carrancho, Eduardo, Larraz Marmol, Bonifacio Cimadevilla Calvo, Carlos Lopez Sanchez, Rebeca Pascual Palacin, Ceferino Osvaldo Perez Pardo, Ceferina Suarez Castano, Raquel Sanchez De Arriba, Ana Solar Herrera.

**Hospital General de Granollers - Barcelona**: Susana Redondo Dia (PI), Regina Sopena, Anna Szatkowska, Maria Pellegrini, Antonia Perez Soto.

**Hospital de la Ribera. Alzira. Valencia:** Sonsoles Aragón Alvarez (PI), Cristina Martinez Escribano, Fernando Sanchez García, Encarna Miñana Aragón, Javier Alonso Garcia, María Gomez Gomez, Jose Emilio Llopis Calatayud.

**Hospital Verge de la Cinta de Tortosa - Tarragona:** Natalia Gallench Pons (PI), Alba Arasa Balaguer, Mireia Armengol Gay, José Luis Barberá Curto, Celia Inés Calderón Coha, Jordi Castellnou Ferre, Anselmo Cortés Benet, Sandra Ferré Almot, Jose Tomás Fos Ortells , Mª Carmen Gomez Martínez, Mercè Mentuy Feixa, Gemma Novas Brach, Antonio Mª Sanchéz Herrero, Antoni Serrat Rabascall, José Tejados Ruiz , Montse Carracedo Mañosa, Caridad Cañadas Morcillo, Vanesa Mascarell Rey, Laia Pla Coto, Sandra Murria Verdera, Maria Vázquez Minguell, Miriam Masià Fumadó, Mª Teresa Arcos Mestre, Mª Carmen Castelló Nicolau, Josep Cabanes Pepiol, Anna Betlem Suárez Bardi, Oscar Colomines Marti, Pilar Beltrol Julian, Mª José Gombau Matamoros, Ivan Pons Moragrega, Lorena Rascón Rojas.

**Complejo Hospitalario Universitario de Pontevedra:** Beatriz Hernandez Vazquez (PI), María de los Ángeles Carro Roibal, Marina Barreiro Torres, Maria Navarro Mateos, Cristina Taboada Barja.

**Hospital Universitario Donostia:** Cristina Gonzalez Serrano (Pi), Berta Castellano Paulis (Pi), Nagore Echeverria Rodriguez, Olga Cengotita Blanco, Silvia Gonzalez Santos, Itziar Olaizola San Jose, Larraitz Aramburu Argandoña, Alazne Enparantza Aiestaran, Nuria Gonzalez Jorrin, Ainhoa Aginaga Badiola, Maria Eizaguirre Cotado, Nerea De La Puente Vitini, Edurne Lopetegui Auzmendi, Amaia Larrea Aseguinolaza, Inmaculada Zubelzu Jaca, Marta Amelburu Egoscozabal, Belen Barandiaran Benito, Claudia Gastesi Larrañaga, Elena Del Val Peciña, Maria Urteaga Aldasoro, Carme Queralt Pascual, Rebeca Alcalde Leon, Maria Toral Cegarra, Ainhoa Garmendia Odriozola, Idoia Gaztañaga Ibarzabal, Iñigo Pajares Merino, Luis Jesus Esnaola Ormazabal, Ane López Montecelo, Marta Aseguinolaza Pagola, Luis Abad Mendia, Amaia Uria Azpiazu, Teresa Gurrutxaga Odriozola, Mª Victoria Arsuaga Olaechea, Ania Casquero Ucin, Olatz Jaca Goena, Lourdes Merino Cadenas, Beñat GarciaGonzalez, Maria Jose Martinez Jimenez, Ana Morales Quiroga, Larraitz Rotetanazabal , Consuelo GarciaVelez, Maria Jesus Goenaga, Edurne Lodoso Ochoa, Beatriz Unceta Barrenechea Orue, Lucia Arnaiz Fernandez, Jose Maria Carrillo Echeverria, Sergio Schillaci Alvarez, , Cristina Garcia Fernandez, Ane Estomba Cabezón,Maria DoLores del Campo Martin, Iciar Ugalde Egaña, M Esther Vicente garcia, Belen Perez Camara, Reyes Ortiz de Urbina, Lucia Arnaiz Fernandez, Manuel Azcona Andueza, Miren Chueca Bolaño, Manuel Eced sanchez,

**Hospital General Universitario Valencia:** Francisco Javier Hernández Laforet (PI), Jose De Andrés Ibañez, Carolina Ferrer Gómez, Carlos Errando Oyonarte, Lourdes Alós Zaragoza, Marta Roselló Chornet., Lucas Rovira Soriano, Elena Biosca Pérez, Andrea Sanchis Veryser, Maria Sempere Mata, Laura Giner Crespo.

**Hospital Son llatzer-Palma de Mallorca:** Maria Dolores Mira Quirós (PI), Jose Luis Aguilar Sánchez, María Inmaculada Valldeperas Hernández, Alisia Cezara Teslev, María Begoña Covas Muñoz, Isabel Fuentes Peña, Rosa María Yáñez López.

**Hospital Fremap Majadahonda - Madrid:** Isabel Cristobal Garcia (PI), Mimosa Hajro Mastori, Francisco José Galindo Sánchez, Eva Abad Fau de Casa Juana, Paula Martinez Fariñas, Rogelio Rosado Caracena, Gabriel Ruiz Córdoba.

**Althaia Xarxa Assistencial Universitària de Manresa - Barcelona:** Francesca Reguant Corominas (PI), Carla Farré Tebar (PI), Marcelo Borderas, Josep Delgado Arteaga, Mauricio Roberrto Argañaraz Quinteros, Isabel Pérez Reche, Consuelo Ruiz Pérez, Meritxell Sabrià Bernadó, Montserrat Cadena Serramitja.

**Hospital Medina del Campo - Valladolid:** Maria Teresa Fernandez Martin (PI), Juan Carlos Alvarez, Beatriz Cano, Christian Bravo.

**Hospital Central de la Cruz Roja San José y Santa Adela – Madrid:** Cristina Stanciu Oana (PI), Sonsoles Martin Alcrudo, Ana Maria Colmenero Ruiz, Cristina Massa Gómez, Carmen Lope Prado, Guadalupe Pobalción Garcia, Alireza Tabatabaian Motamendi, Julio Aitor Garcia Matas, Alexandra Smaranda Andonie, Maria Mata Diaz, Luis Fernando Simon Cirujano.

**Hospital Universitario Río Hortega - Valladolid:** Jesús Rico Feijoó (PI), Cesar Aldecoa, Yessica Guerra Restrepo, Alba Herrero Garcia, Sara Pelegrin, Alicia Bordell, Irene Lopez Catalan, Clara Bolaños, Borja Morales Jaquete, Rocio Rioja Garrido, Mario Madrid Tribano, Itziar Mendez, Silvia Montero Caballero.

**Hospital Universitario Ntra. Sra De Candelaria. Santa Cruz De Tenerife:** Elena Espinosa Domínguez (PI), David Domínguez García, Israel Amador García, Ana Belén Fernández Pérez, Gabriela González, Raúl Hernández Bishop, Samuel Hernández González, Teresa León SanSegundo, Lucía Pazos Otero, Marta Pérez Méndez, Carolina Ramos Montero, Irina Rodríguez, Marina Sánchez Navas, Luis Soto Jáquez, David Viera Camacho.

**Hospital Universitario de Canarias:** Luis Manuel Pérez Guillama (PI), María Esther Herrera Piñero, Hugo Rosero Rosero, Pablo Marín Serralvo, Antonio Rodríguez Medina, Ana María León Fragoso, Vanesa González Fariña, María del Carmen Martín Lorenzo, Jorge Solera Marín, Jessica Hernández Belsmeisl, Beneharo Darias Delbey.

**Hospital Universitari General de Catalunya - Sant Cugat del Vallés - Barcelona:** Núria Pesa Vendrell (PI), Julián Roldán Osuna, Clara Bordes García, Gabriela Altamirano Grimaldo.

**Hospital Universitario Virgen Macarena - Sevilla:** Estefanía Peralta Espinosa (PI), Aurora Cruz Ortega (PI), Antonio Ontanilla López, José Manuel Prieto Gutiérrez, Bartolomé Fernández Torres, María de los Ángeles Ariza Fernández, Héctor Berges Gutiérrez, Fernando Gónzalez Yanes.

**Hospital General Universitario José María Morales Meseguer- Murcia:** Petra González Pérez (PI), José Antonio Castillo Bustos, Mª del Mar Serna Barquero, Ana Belén Alcaraz Martínez, Mercedes Benitez Jiménez, Julia Mula Leal, Cristina Díaz Fuentes.

**Hospital Francesc de Borja de Gandía - Valencia**: Eva María Gimbert Burgos (PI), Noemí Almenara Almenara, Belén Bardisa de la Iglesia, Aída Benlloch Beitia, Waldemar Dabrowski, Agustín Guerri Cebollada, Gregorz Kwiatkowski, Vicenta López Rubio, Sofía Machado Ortíz, Amanda Miñana Moll, Magdalena Agnieszka Pasek, Juan Baltasar Penella Ferra, Carmen Rizo Martí, Mª Ángeles Soldado Matoses, Elena Clara Casado Pineda, María Alexandra Korobkoff Fernández.

**Hospital Universitario Álava:** Margarita Logroño Ejea (PI), María Carmen Iturricastillo Pérez, Erika Olea De la fuente, Ibai Iriarte Zaranton, María Gastaca Abasolo, María José Muñoz Sanz, Ana Mendiguren Murua, Rosa Sarachaga Isasi, Alaitz UrtiagaUrrestizala, Gorka AldalurMuxika, Ane Errasti Iturrizar, Ania Albinarrate Fernández, Miren Agurtzane Jauregui Barrenetxea-Arando, Diana María Rodríguez Sanabria, Lucía Pérez De AlbenizVesga, Ramón De Luis Casis, María Rosa Ramos Vega, Ana Soto Iglesias, Ana Fondarella Sales, Begoña Rementería Castillo, Begoña Pérez De San Román Basabe, Francisco Borja Barrachina Larraza¡, Marta Pérez González, Asier Atxa Guinea, María Belén Beltrán De Heredia Pérez De Villareal, Aitor Martín Rodríguez, Ana Ugarte Mieres, Luis Francisco Gutiérrez Sánchez, Nagore Saez De Ibarra López, Miren Idoia Aguirre Oteiza, Javier Estalayo Rodríguez, Carla Rosario Houghton Acuña, María Del Carmen PavesioYusta, Fidel De Celis Gutiérrez, Cristina Carmona Lana, Nieves Ortiz-Roldán Rodríguez, Juan María Marcos González.

**Hospital Alto Deba-Arrasate-Mondragón:** Pablo Renedo Corcóstegui (PI).

**Hospital de Zumárraga, OSI Goierri Alto Urola:** Sandra Liliana Muñoz Pérez (PI), Iñaki Roa Martinez, Maria Jesús Madina Albisua, Angel Beriain Arcelus, Edurne Gaona Fernández, Maitane Rubio de Jesús, David Castillo Gómez, Marco Amambal Altamirano, Carlos Enrique Valladares Hernández, Inazio Zearreta Letamendia.

**Hospital Universiari Joan XXIII Tarragona:** Eva Ferreres Albert (PI), Judit Saludes Serra, Paula Arauzo Casedas, Luís Carrillo Luna, Diego Prendes Fernandez.

**Hospital Sant Joan D´Espí Moisès Broggi - Barcelona:** Alex Romero Fernández (PI), Sira Garcia Aranda, Miroslawa Konarska, Meritxell Serra Valls, Maria Jose Bernat Alvarez, Jesus Fernanz Anton, Maria Carmen Deiros Garcia, Olaia Guenaga Vaqueiro, Sandra Marmaña Mezquita, Ana Tejedor Navarro, Laura Grau Torredeflot.

**Centre d'Atenció Integral Dos de Maig - Barcelona:** Patricia Margalló Zapater (PI), Josep Masdeu Castellví, Carme Deiros, Gloria Molins, Agnieszka Golska, Sandra Marmaña, Cesar Gracia, Ruben Chacon, Meritxell Serra, Pere Serra, Laura Grau.

**Hospital General Universitario Elche - Alicante:** Ana Pérez Carbonell (PI), Dr. Enrique Cantos Gómez, Jaime Miralles Sancho, María Mercader Alarcon, Dianan Berrio Grajales, Elena Miranda Tauler, Marta Resalt Pereira, Natividad Más, Francisco Guillén, Vicent Cuquerella, Carlos Picó, Adolfo Morente, Javier Solis, Javier Gil.

**Hospital de Sagunto - Valencia:** Alicia Sanchez Hernandez (PI), Julio Llorens Herrerías , Maria Josefa Gimeno Campos, Raúl Duplá Pérez.

**Hospital Universitario Doctor Peset - Valencia:** Joana Baldó Gosàlvez, Juan Vicemte Llau Pitarch, Marta Beatriz Navas Moruno, Jenny Katherine Medina Vasques, Esteban Rodríguez Ortiz.

**Hospital Universitario Quironsalud Madrid:** Rocio Díez Munar (PI), Jose Maria Marzal Baro, Ines Garcia Barrasa, Daniela Cubek.

**Hospital Juan Ramón Jiménez – Huelva:** Maria De la Peña Gómez Dominguez (PI), José Ernesto Gallo Mena, Alejandro Gavira Leon, María Dolores Díaz Lara, Pablo Longo Guridi, Ana Mª Quintero Moreno , David Soriano Lopez, José González González, Francisco Romero Caro, Irene Mojarro Zamora, Juan Victor Lorente Olazábal, José Manuel García Garcia, José Luis Bonilla Garcia, Cecilia Prieto Candau, Javier García Andreu, Marisol Hernández del Castillo, Ignacio Algarín del Campo, Irene Jarana Aparicio.

**Hospital Lluis Alcanyís de Xàtiva – Valencia:** Esperanza Fernandez Bañuls (PI), Vicente Domingo Triadó.

**Complejo Hospitalario Universitario Insular Materno Infantil - Canarias:** Aurelio Eduardo Rodríguez Pérez (PI), Elisabet Guerra Hernández, María Luisa Torres Machín, Arantxa del Carmen Ramos Álamo, Zoraya Hussein Dib González, Roberto Fariña Castro, Víctor Daniel Betancor Lindström.

**Hospital Universitario La Paz - Madrid:** Alejandro Suárez de la Rica (PI), Laura Ciudad Morales, Claudia Cuesta González-Tascón, Emilio Maseda Garrido , Emilia Guash Arévalo, Elena Alvar Palanco, Teresa Prim Martínez, Mercedes Campos Sanz, Alicia Rubio Sánchez, Eduardo Alonso Yanci, Elena Cortés Fornieles.

**Hospital Universitario Reina Sofía - Córdoba**: María Concepción Ruiz Villén (PI), Juan Jose Cidoncha Rodriguez, Arantxa Rodriguez Jimenez, Jesús David Rubio López, Teresa Gonzalez Espinosa.

**Hospital Clinico Universitario de Valencia:** Ferran Serralta Cabero (PI), Marina Soro Domingo, Francisco Javier Belda, Mario de Fez, Ana Jurado Pulgar, Esther Romero Vargas, Ferran Serralta Cabero, Gerardo Aguilar, Gemma del Castillo, Blanca Aroca, Ernesto Pastor Martínez, Sara martínez , Laura García Vargas, Luis Belmonte, Javier Cuervo, Elizabeth Bárcena.

**Hospital Doctor Jose Molina Orosa. Arrecife , Lanzarote:** Juan Ruiz Canton (PI), Maria Jose  Borrego Pedrera, Yesica Sanfiel  Diaz, Armando Hernandez  Martinez.

**Hospital Ruber Internacional-Madrid:** Daciano Álvaro Gaona Atienza (PI), Marco Antonio Taboada Gomila, Javier Benito Martínez, Pedro Santos Sogo, Pilar Bernad González, Juan Pablo Von-Walter, Isabel Gómez García, Ana Moreno Sánchez, Octavio Orte Aldea, Álvaro Elicegui Ortiz, Uxío García Aldao, Álvaro De La Vega Terol, Judit Benítez Villar.

**Hospital Universitario Guadalajara:** Samuel Martin de la Vega (PI), José Ramón Rodriguez Fraile, Raquel Muñoz Expósito, Victor Jimenez Guitián, Zully Cristina Benzo Aguilera, Carlos Galan López.

**Complejo Hospitalario Universitario A Coruña:** Marta Vidal Seoane (PI), Felisa Álvarez Refojo, Sonia Gonzalez Bardanca, Anxo Vilar Castro.

**Hospital Mancha Centro-Alcazar de San Juan. Ciudad Real:** Begoña Gonzalez Molina (PI), Pedro Cuesta Santos, John Carlos Perez Moreno, Anselmo Martínez Blázquez, Francisco Quezada Moreno, Roberto Antonio Muñoz Acosta, Rosa Maria Morenza Perez, Susana Claudios Santana, David Navas Manchado, Javier Cruz Tejado, Almudena Fradejas Lucas.

**Hospital Central de La Defensa Gómez Ulla – Madrid:** Ricardo Navarro Suay (PI), Miguel Angel García Aroca.

**Complejo Hospitalario Universitario de Badajoz-Badajoz:** Juan Ricardo Caro González (PI), Fernando Sánchez Espinosa, Isabel Alejandra Becerra Cayetano, Laura Laso Ramírez, Cristina Pinilla Silva, Laura Caperote Sanchez, Ana Marín Moreno, Darío Carvajal Muñoz, Beatriz Pilo Carbajo.

**Hospital Parc Tauli.Sabadell - Barcelona:** Mercedes Rosas Lario (PI), Carmen Colilles Calvet, Marta Barquero López, Anna Artigas Soler, Andrea Isabel Vallejo Tarrat, Carolina María Yepes Fernández, Clara Pilar Sáez Ibarra.

**Complejo Asistencial Universitario de León:** Ana María Pérez Villafañe (PI), Juan Carlos Bermejo Gonzalez, Ana Isabel Fernandez Díez, Cristina García- Miguel Sanchez, Ana Martín García, Sofia Nicolas Aller, Beatriz Blanco Cuevas, Ana Belen Rubio López, Consuelo Rego Díaz, Rodrigo Pérez Blanco, Concepción Marín -Blázquez Montiel, Silvia Ferrer Cerón, Susana Blanco García, Fernando Díez Burón, Alicia Alonso Cardaño.

**Hospital Santa Creu i Sant Pau - Barcelona:** Jose Maria Gil Sánchez (PI), Maria Victoria Moral García, Astrid Batalla, Marta Giné, Alfred Merten, Luisa Cueva.

**Hospital Universitario de la Princesa - Madrid:** Mar Orts Rodriguez (PI), Antonio Planas Roca, Carlos Figueroa Yusta, Fernando Ramasco Rueda, Rosa Mendez, Esperanza Mata Mena, Jesús Nieves Alonso, Sheila Santidrian, Carmen Rodríguez Garcia, Olaya Alonso Viejo, Juan Alvarez Pereira.

**Hospital General Universitario de Alicante:** Carlos Ferrero Coloma (PI), Luis Gómez Salinas, María Galiana Ivars, Eva Such Camargo, Ana Fuertes Olivera, Pilar García Segura, Clotilde Funes Moreno, Úrsula Toral Toral, Belén Rodríguez Mas, Ana Rosa Martínez Ibáñez, Melodie Álvarez Garoña, Santiago Pardines Rico, Josué Martínez Vela, Vicente López Gil, Joaquín González Fernández, Luis González Jiménez.

**Hospital de Mérida - Cáceres:** Jose Maria Tena Guerrero (PI), Enrique González, Estefanía Palma Gónzales, Enrique  Gónzalez Sánchez.

**Hospital Universitario Puerta de Hierro Majadahonda - Madrid:** Luisa María Glez Pérez (PI), Javier García Fernandez, Beatriz Alonso Menárguez, , Ana Alvarez Bartolome, Natalia Muñoz Ávalos, , Maria Casado Salcedo, Alejandra, Del Campo Mur, Sara Del Valle Quintans, Cristina Ferreras De La Riva, Inocencia Fornet Ruiz, Cristina Garicano Madrigal, Luisa Maria Gonzalez Perez, Ana, González Roman, Lucia Hormaechea Bolado, Jose Ignacio Lora Tamayo, Antonio Manzano Rodríguez, Olga Martín García, Fátima Martinez Jimenez, Viktoria Molinar, Inmaculada Mourelle González, Ana Isabel Peral Garcia, Rodríguez Marta Manzaneque García - Alcañiz, Guillermo Rubio Espinosa, Belén San Antonio San Roman, Rocío Segovia Martínez, Roberto Siljeström, José Alberto Suarez Del Arco, José Luis Belmonte Gago, Verónica Guilló Moreno, Cristina Sánchez Gonzalez, Paula Rey Jiménez, Reyes Iranzo Valero, Teresa Torre Oñate, Jessica García Suarez, Mercedes Vidal Fernandez, Nadia Akram Abdallah Kassab, Oscar Sánchez López, David Sánchez Pérez.

**Hospital Arnau De Vilanova-Lliria. Valencia:** Vicente García Ferrando (PI), Sara Alegre Ferri, Estefanía Boix Tundidor, José Luis Carrión García, Juan Carrizo Sánchez, Jaime García Ramón, Carlos García Marquez, Miguel Angel Gil Company, Ana Herrero Miguel, Enrique Lloria Pons, Concepción Peña Roselló.

**Consorci Sanitari del Maresme, Hospital de Mataro- Mataro - Barcelona:** Elisa Reñé de Antonio (PI), Montse Yuste Graupera, Raquel Algilaga Segovia, Isabel Fábregas Blanco, Sergio Vitale, Alexia Nebot Galindo, Marta Villanova Baraza, Casandra Gimeno Grauwinkel, Juan Camilo Meza.

**Hospital Universitari Mútua Terrassa - Barcelona:** Pablo Alcántara Pinillos (PI), Carme Pérez, Mònica Pérez Poquet, Magín Morales Morales, Alba Cebrián Moreno, Aurora Rodríguez Campos, Marga Novellas Canosa, Olga Gómez Ortiz, Miguel Marín Moya.

**Parc Sanitari Sant Joan de Déu, Sant Boi de Llobregat - Barcelona:**  Laura Santos Sánchez (PI), Marta Sánchez Martín, Meritxell Ojer Catalán, Caridad Estrella Torrens Hernandez, José Giménes Crouseilles, Gregory José Contreras Pérez, Pablo Martinez Barabino, Paulina Camarena Palacios Maceda, Eva Digón Molina, Diana Irene Campello Ros, Carmen Luisa Rodriguez Pérez, Dionilux Gamero Acosta, Bertha Liana Martinez Cotrina, Samuel Vicente González Martin, Ariadna Tuya Calvo, Pere Estivill Rodríguez . Hospital Don Benito: Marta Lopez Marqués, Enrique Del Cojo Peces, María del Pilar Rodríguez Chaparro.

**Hospital Universitario Severo Ochoa Leganés, Madrid:** Marta Vicente Orgaz (PI), Gema Fraga Casais, Cristina Crespo Gómez, Yasmina Gonzalez Salvador, Carmen Pérez de Isla, Adela Gardeta Pallares, Clara Fernandez Sánchez, Adriana Orozco Vinasco, Purificación Sánchez Zamora, Gema Márquez Garrido, Raquel Gonzalez Velasco, Juan José Correa Barrea, Soraya Gholamian Ovejero, Blanca Gómez del Pulgar, Belén Rodriguez Sánchez- Cabezudo, Rubén Saz Castro, Sonia Sanchez Solano, Alejandro Zamora García, Antonio García Rueda, Rossel Alina Mejía Arnau.

**Hospital General de L'Hospitalet - Barcelona:** Maria Sanabra Loewe (PI), Vanesa Tejedor de la Fuente, Adriana Aponte Sierra, Carlos Ardila Olmos, Natalia Montero Gaig, Ana Maria Vicol, Gisela Egido Lopez.

**Hospital Universitari i Politècnic La Fe - Valencia:** Salomé Matoses Jaén (PI),  Lucía Blanca Cabezudo de la Muela, Rosario Vicente Guillén, Mª José Alberola Estellés, Paula Pérez Caballero, Marysol Echeverri Vélez, Trinidad Simó Cortés, Laura Angelina Bruno Carlos, Amparo López Gómez, Esther Pérez Sancho, Francisco Femenia Price, Begoña Ayas Montero, Raquel Ferrandis Comes, Laura Mínguez Luján, Silvia Polo Martínez, María Batista Doménech, Ignacio Albero Roselló, José García Cantos, Pilar Argente.

**Hospital Universitari Bellvitge - L´Hospitalet - Barcelona:** Maria José Colomina (PI), Esther Méndez, Albert Gil Dorado, Inmaculada Camprubí , Montserrat Mallol, Marta Costa, Victor Caño, Javier Bocos, Miguel Ángel Delgado, Laura Contreras, Albert Pi, Vinyet Lopez, Antonia Bonet, Marta Caballero, Irene Macia, Marc Gimenez , Maylin Koo, Anderson José Vergara, Laura Viguera, Lourdes Pérez, Ana Vasquez, Sheila Solsona Jordi Xicola, Guillermo Alonso, Gerard Turmo, Guillermo González, Roser Bayona, Sara Garcia, Peter Adamove, Adriana Rueda, Carolina Vieito, Guillermo Puig, Elena Campistol, Ely Jenssen, David Alvarez, Raul Herrera, Antonio J Navarro, Onésimo Alaniz, Xavier Balderas, Antoni Sabate Pes.

**Hospital Universitario Cruces - Bilbao:** Fernando Iturri Clavero (PI), Alberto Martinez Ruiz, Estibaliz Bárcena Andrés, Oscar Prieto Calderón, Maite Elguezabal Laucirica, Mº Eugenia García Durruti, Gonzalo Tamayo Medel, María L. Hernández Sanz, Celia González Painagua, Andrés Álvarez Campo, Gorka Ojinaga García, Mireia Pérez Larrañaga, Julia T. Herrera Diez.

**Hospital Universitario Príncipe De Asturias - Alcalá De Henares, Madrid:** Fátima Cañeque Yanini (Pi), Maximo Sanz García, Cristina Lasa Unzue, Luis Bejarano Redondo, María Ángeles Mancebo Zafra, Daniel Marivela Palacios, Raquel Rodríguez Guerrero, Manuel Herrera Bach, Raquel Chaves Rodriguez.

**Hospital Universitario Infanta Leonor – Madrid:** Eugenio D. Martinez Hurtado (PI), Eugenio Martínez Hurtado, Alfredo Abad Gurumeta, Norma Aracil Escoda, Beatriz Nozal Mateo, Elena Lucena de Pablo, Elena Nieto, Ana Nieto, Bárbara Algar Yañez, Elena Sáez Ruiz, Kateri Chao Novo, Javier Ripollés-Melchor, Alicia Ruiz Escobar.

**Fundación Jiménez Díaz- Madrid:** Paloma María Santiago Paniagua (PI), Luis Enrique Muñoz Alameda, Patrícia Mellado Miras, Ana Zapardiel Lancha, Mónica Giménez Hernández.

**Hospital General Universitario Virgen de la Salud, Elda- Alicante:** Cristina Embid Roman (PI), Calixto A. Sánchez Pérez, Mª Victoria González Latorre, Ana Martínez Gil, Cristina Pastor Lain, Mª Carmen Ribera Montés, Pablo Asensi Iniesta, David Ivorra Morell.

**Hospital Rey Juan Carlos, Mostoles - Madrid:** Mercedes Itza Barranco (PI), Andrés Gómez Olmedo Rosado, Concepción Camus Sanchez, Jaime Bragado Domingo, María Cruz Baquero, Juan Carlos Estupiñan Jimenez, Jose Luis Ayala Ortega.

**Hospital Vall d´Hebron - Barcelona:** Marisa Paños Gozalo (PI), Inmaculada Vives Llorente, Sara Bóveda González, José Mª Nieto Rodríguez, Mª Dolores Mateo Arzo, José Antonio Jiménez Pérez, Jesús Cirbian Franqueira, Sonia Nuñéz Aguado, Fernando Clau Terré, Inmaculada Salgado Algaba, Rosa Antúnez Elvir, César Botana Sicilia, Gemma Usúa Lafuente, Esther Ciércoles Jiménez , Cindy Mokund González, José Caldentey Sierra, Cristina Diaz Amaral, María Iborra Escalona, Lidia Mora Miquel, Encarnación Guerrero Viñas, Hector Torres Rios, Carlos Salvadores de Arzuaga, Marta Juste Lucero, Cristina Clara Esquerré Hernadez, Maria Consuelo Moyano Fernandez, Verónica Estepa Calvo, Joan Pla Bosch, José Manuel Naya Sieiro, Anna Server Salvà, Patricia Guilabert Sanz, Lucia Porteiro Mariño, Vanessa Sanchéz Torrents, Erika Schmucker Agudelo, Sonia Francés, Anna Conesa Marieges , Irene García Martínez, Verónica Alonso Mendoza, Sandra Galve Navarro, Eva Andreu Riobello, Montserrat Feliu Lloret, Nuría Montferrer Estruch, Elena Suarez Edo, Rosario Nuño Sanz , Anna Abad Torrent, Manel García Gorriz, Mª Carmen Suescum López, Pilar Cortiella Garreta, Elena Vilardell Ortiz, Ivan Villaverde Castillo, Montserrat Pascual Arellano, Cristina Muro Castro, María Marín, Alfons Biarnés Suñé, Hector Duque Santos, Marcelo Alejandro García, Miguel Ángel González Posada, Susana Manrique Muñoz, Susana González Suárez, Patricia Bascuñana Fornells, Serafín Alonso Vila, Laura Llinares Espí, Helena Serrano Tuero, Victor Martin Mora, Mª Pilar Tormos Pérez, Victor Morales Ariza, Yuri Loaiza Aldean, Ana Rodriguez Tesouro, Esther Cano Peral, Eva Mª Pelaez de la Fuente, Gloria Agreda Martínez, Patricia Galán Menéndez, Julio Antonio Meza Vega.

**Hospital Universitario Virgen de la Arrixaca. El Palmar: Murcia:** Jesús López Pérez (PI), Francisco Acosta Villegas, Maria Espinosa Aguilar, Jesús López Pérez, Joaquín García Ferreira, José Antonio García López, María Piedad Martínez Gil, Luis Falcón Araña, Antonio García Candel, Javier Belmonte Justamante, Fabián Esteban Vittortas Firganek, V. Raquel López López, José Antonio García Martínez, Mireya Arnez Paredes, Cristina Muñoz García, Natalia Villa Lorarte, Bárbara Lorca Martinez, Marcos Salmerón Martín, Pedro Torres Mosquera, Javier López Ayala, María Dolores Riquelme Contreras, Giovanni Alessandro Ercole, Jorge Sánchez Lozano, Celia MiñaNo Frutos, Mar Vidal Almela, Fernando Martinez Juesas, Marina Diez del Hoyo, José Hurtado Marín, Ana Sánchez Amador, Máximo Torres Ganformina, Ana Bastida Chacón, Luis Enrique Fernández Rodríguez.

**Hospital Clínico Universitario de Valladolid:** Maria Pérez Herrero (PI), Jose Ignacio Gómez Herreras, Felipe Muñoz Zurdo, Iciar Martínez Almeida, Pilar Olmedo Olmedo, Angel Fernández Collantes, Gema Rodríguez Cerón, Rita Pilar Rodríguez Jiménez, Alfredo Carrera González, Maria Teresa Pelaez, David Velasco, Henar Muñoz, Pilar Rubio Babiano, Elisa Alvarez Fuente, Gabino Mozo Herrera, Francisco Javier Lomo, Andrea Sánchez Miguel, Julia Martín Romo, Cecilia Bartolomé Bartolomé, Alejandra Fadrique Fuente, Alma Vallejo Gago.

**Fundació Puigvert, Barcelona:** Pilar Sierra Arnedo (PI), Sergi Sabaté Tenas, Daniel Hernando Pastor.

**Hospital Universitario Miguel Servet, Zaragoza:** Concha Cassinello Ogea (PI), Salvador Laglera Trebol, Juan Pablo Ortega, Marta Infantes Morales, José María Remartínez, Inma Gonzalo, Luisa Lacosta Torrijos, Alejandra García Hernández, M.ª Pilar Herranz Andrés, Concepción Fraca Cardiel, Enrique Latorre Marques, Consuelo Gomez, Marcos Polo Pellicena, Ramon Alias Gomez, Coral Rodriguez Calvo, Roberto Carbonell Bernal, Roberto Gómez, M.ª Concepción Pardillos Calatayud, Leticia Oliden Gutierrez, Inmaculada Gonzalo Pellicer, José Viñuales, Cristian Aragón Benedí, Fernando Gil Marín, Pilar Forcada Vidal, Sara Visiedo, Alejandro Gracia Roche, Francisco Jiménez Riera, Pablo Oliver Forniés, Marimar Pérez Morente, Berta Pérez Otal, Yolanda Durán, Andrés Millaruelo Ramí, Alejandro Lucas luelma, Paula Jarén Cubillo, Ana Ramón Navarro, Beatriz Simón Rivero, Lucía Tardós Ascaso, Cristina Bueno Fernández, Fernando Carbó Espinosa, Oscar Urbano Gonzalo, Ana Asensio Paris, Paco Sandín, Adela Ruiz de la Cuesta, Pilar Juvera, Marian Ortega, Lucia Gallego Ligorit, María Puertolas, Jorge Valles, Irene Molinos, Borja Rubio García, Nacho Cotera, Blanca Izquierdo Villarroya, Alberto Sanjuan. Hospital Reina Sofía, Tudela: Sandra Marcela Gil Caballero (PI), Jose Manuel Vicente de Vera Floristan, Ernesto Fernández Basterra, Jaume Riú Pelegrí, Juan Ros Añón, Antonio Manuel Martín Rubio, Ignacio Armendáriz Buil, Fernando Larrodé Pastor, Josu del Río Manterola, Mónica Elena Vargas Cerdán, Laureano Menéndez Ozcoidi, Pablo Baltanás Rubio.

**Complejo Hospitalario Torrecárdenas - Almería:** Jonathan Estévez Santiago (PI), José Santiago Martín, María Ricart Asensi, Javier Fernández Méndez, Ana María Prieto Cabrera, Isabel Navarro Méndez , María del Rosario Blanque , Milagros Aguado Frutos, José Antonio Delgado Tapia, Wilsiane Amaral Calvante, Irene Jiménez Ayala, Elisabet Reche Navarro, Rocío Rodríguez Contreras, Rafael García Herrero, Carmen María Muñoz Corchuelo, Nicolás Burgos Gabriele, Desiré Agudo Ponce, Sofía Bes Miras, Jose Joaquín Arjona Arjona, Lilia Regina Bernal Basurto, Encarnación Cánovas Fernández, Eva María García Fuentes, Manuel Cortiñas Sáenz, Ana Isabel Navajas Gómez de Aranda.

**Hospital Obispo Polanco, Teruel:** Julia Ungría Murillo (PI), José Antonio Ruiz Doñate, Gustavo Adolfo Armas Tova, Fernando Murciano García, Nayra Lupe Nina Colque, Rebeca Alonso Salas, Patricia Tena Galindo, Elsie Cañizares Mota.

**Hospital Universitario Virgen de la Victoria de Málaga:** Jose Cruz Mañas (PI), Aurelio Gómez Luque, Enrique Sepulveda Haro, Salvador Romero Molina, Maria Soledad Bueno Garcia, Juan Carlos Molina Ruiz, Manuel Baena Lopez, Aida Raigon Ponferrada.

**Hospital Plató, Barcelona:** Andrés Gallego Ledesma (PI), Maria LLuisa Moret Ferron, Victor hosta Mateu, Maria José Miro Domenech, Olga Alemany Vilalta, Sergi Viedma Crespo, Consuelo Congo Silva, Oscar Salmeron Zafra, Marcela Ceballos Burbano, Teresa Garcia Navia, Grover Vila Rubina, Isabel Pons Casanovas, Juan Antonio Mani Ibañez.

**Fundació Hospital Sant Joan de Déu, Martorell - Barcelona:** Roser Creixell Busquet (PI), Luís Muñoz Falcón, Luis Alberto Abadal Borges, Sonia Carrera Rubio, Carlos Castro Barreda, Gerytza Chagas de Alcántara, Albert Codina Pujol, Carlos Garcia Abascal, Javier Martínez Milán, Alejandro Mercero Domínguez,  Danitza Silva Pérez.

**Complejo Hospitalario de Jaén:** Sonia Gutierrez Jimenez (PI), Juan Manuel Ortiz, Alejandro Martínez García, Enrique García Vena, Almudena Morales Rojas, Laura Rodríguez Melguizo, Ángela María Soriano Pérez, María Eugenia Navío Poussivert, Ana Centenera Sánchez, Laura García Siles, Mercedes Poncela García, María Paz Béjar Palma, Magdalena Martín Ortíz, María Pilar Millán Bueno, Miguel Angel Díaz Expósito, Estefanía Peña García, Ana Pineda Muñoz , Álvaro de la Torre Giménez, Felipe García Chacón, Luis Gonzalez Vicente, Fernando Rámila Fernández de Soria, Vito Mario Fernandez Bullejos, Raquel Ramos Lozano.

**Hospital Virgen de la Salud – Toledo:** Mª Sherezade Tovar Doncel (PI), Filadelfo Bustos Molina, Daniel Paz Martín, Jesús Porro Hernández, Mª Paloma Poza Hernández, Bárbara Vazquez Vicente, Pilar Adán Valero, Cristina González Lantero, Mª Victoria Fernández-Roldán Galán, Mª Jesús Domínguez Bronchal, María Jesús Martín Jiménez, María Teresa Bienciento Murga, Beatriz Romerosa Martínez, Sara Resuela Jiménez, Paula Álvarez Buitrago, Lorena Calvo Frutos, Paloma Herrador Iradier, Beatriz Castaño Moreira. Hospital Clinico Universitario Lozano Blesa: Beatriz Fornies Gimenez, Beatriz Ruiz, Pilar Aguelo, Esther Cuesta, Ester Polo, Monica Araujo, Maria Badel, Marta Pedraz, Beatriz Navarro, Galo Baclini, Patricia García, Aurora Callau, Laura Pradal, Maria Carbonell, Belen Albericio, Mario Lahoz, Sergio Gil, Laura Fores, Julia Guillén Antón.

**Hospital Universitario Arnau de Vilanova, Lleida:** Johana Mercado de la Cruz (PI), Laura Urbano Arcila, Sandra Rodriguez Calvo, Alejandro Pérez Requena, Marijke Sneyers Closa, Sara Sanchez García.

**Hospital Universitari Josep Trueta, Girona:** Judith Lopez Fernandez (PI), Berta Baca Pose, Neus Sargatal Borell, Fina Parramón Vila, Maria Díaz Martínez.

**Hospital Universitario del Vinalopó - Alicante:** Clara Diaz Alejo Marchante (PI), Diego Garcia Gerona, Jorge Tadeo Puchol Castillo, Antonio Fernandez Casares, Jaison Pio Silva.

**Hospital Mutua Accidentes de Zaragoza:** Clara Marín Zaldívar (PI), Antonio Urieta Carpi, Jose Ferrandez Arenas, Amagoia Fernandez de Gamarra Goiricelaya, Carmen Teixeira Almarza.

**Hospital Provincial Nuestra Señora de Gracia, Zaragoza:** Juan R Marin Guerricabeitia (PI).

**Hospital Ernest Lluch Martin, Calatayud - Zaragoza:** Mara ortiz (PI), Maximiliano Toro, Silvia Martínez, Leticia Pérez, Ana Garcia, Laura Carnicero.

**Hospital Quiron Bizkaia:** Meli Fernandez Mencia (PI), José luis Alcibar, Noelia Martínez Villanueva, Veronica Alcayaga di Palma.

**Fundación Hospital Espíritu Santo, Barcelona:** Encarnación Peral Molina (PI), Pere Ortells Nebot, Gloria Lucas Domingo.

**Hospital de Alcañiz - Zaragoza:** Carlos David Albendea Calleja (PI), Patricia Calderón Aguirre, Patricia Edith Calderón Aguirre, María Cristina Carluccio, Jesús Garafulla García, Raquel Murillo Pina, Elisa Montón Millán, Teresa Marín Abad, Carmen Bona Gracia.

**Complejo Asistencial Universitario de Salamanca:** Laura Nieto Martín (PI), Jose Mª Calvo Vecino (PI), Mario Vaquero Roncero, Elisa Sánchez Barrado, Mª Mar Fernández Campos, Miguel V. Sánchez Hernandez, Valentín Javier Santana González, Teresa López Correa, José Alfonso Sastre Rincón, Antonio Rodriguez Calvo, María del Pilar Arribas Pérez, María Eugenia González Algarra, Carolina Jambrina García Montoto, Eduardo Sánchez López, Maria José Villoria Medina, Ana Vara Miranda, María Jesús Pascual Lorenzo, María Isabel Martínez Trufero, Pablo Alonso Hernández, Manuel Jesús Sánchez Ledesma, Jose Carlos Garzón Sánchez, Pilar Sánchez Conde, Domingo Bustos García, María Isabel de Celis Jiménez, Isabel Pingarrón Hernández, Gonzalo García Benito, Agustín Díaz Álvarez, Gerardo Riesco Galache, José Ángel Sánchez Crespo, María Heredia Rodríguez, Virgilio Emiliano Martín Vicente, Carlos Javier Espinel Olanda, Jose Luis González Rodríguez, Lucio San Norberto García, Gemma Yusta Martín, María Isabel Garrido Gallego, María Azucena Hernández Valero, Alberto de Diego Fernández, Rosa María Prieto Martín, Juan Ignacio Santos Lamas, Daniel Salgado García, Alberto Rios Llorente, Jordanna Almeida cristo Barbosa, Sonia Iolanda Freire da Silva Moreira, Ignacio Trejo González, Mercedes Rodríguez Rojo, Diego Pérez Cáceres, Stephanie Pamela López Vega, Diego Luis Leoz Taboada, Elisa Jausoro Saracho, Silvia Casas Ramos, Ainhoa Rivero Martín.

**Hospital Universitario La Moraleja - Madrid:** Roberto Ruiz Abascal (PI).

**Complejo Hospitalario Universitario Insular-Materno Infantil de Las Palmas de Gran Canaria:** José Valín Martínez (PI), Yeray Rosario Auyanet Déniz, María Dolores Betancort Gutiérrez, Lourdes Hernández González, Oliver Monzón Déniz, Jesús Martínez Sopena, Janet Quintana Sánchez, Rut Aracil Gonzalez, Agustín Fuentes González, Abraham Ortega Ramos, Ana Lucía Cervantes Valdivia, Manuel Mañas, Arimar Alonso Santana, Marta González Navarrete, David Gajate, Gloria Cejas Marmol.

**Hospital Regional Universitario de Málaga:** Alejandro Barroso (PI),Juan Carmona Aurioles, Jose Maria Laza Reín, Fernando Santos, Maria Valencia, Marina Ruiz Palomo, Nathanael Knowlson, Antonio Milla.
