# Supplementary material for: Observational study on fluid therapy management in surgical adult patients
Source: BMC Anesthesiol. 2021 Dec 13;21:316. doi: 10.1186/s12871-021-01518-z (PMC8667365; doi:10.1186/s12871-021-01518-z)
Supplement: Supplementary file 4 — Additional file 4. [file 12871_2021_1518_MOESM4_ESM.docx]

**Highlights – Fluid Day Study**

- The Fluid Day study found that balanced crystalloid and normal saline 0.9% are the most widely used intravenous fluids in any surgery in most of the patients.
- In more than half (54%) of the high-risk patients and/or high-risk surgeries, an average of 2 types of crystalloids was administered, of which 1 was normal saline 0.9 %.
- Fluid Day showed that the total volume/kg/hr administered in the intraoperative period was lower in patients with high risk.
- Only 15% of patients undergoing high-risk surgeries included in Fluid Day underwent advanced haemodynamic monitoring, and less than 10% received goal-directed therapy.
- The Fluid Day study gives an overview of the anaesthesiologist’s attitude towards different anaesthesia and surgical risks. This is the first observational study of real fluid therapy management in Spain.
- We did not link the variability observed in our study with any postoperative results. Although this is of interest for future studies, we believe that the existence of such variability is in itself an important issue to address in order to improve the quality of fluid therapy management.
